# Imaging intracellular zinc by stimulated Raman scattering microscopy with a small molecule vibrational probe

**DOI:** 10.1039/d5sc03442f

**Published:** 2025-11-04

**Authors:** Elsy El Khoury, Symara de Melo Silva, Naixin Qian, Vinh Gia Vuong, Wei Min, Daniela Buccella

**Affiliations:** a Department of Chemistry, New York University New York NY 10003 USA daniela.buccella@emory.edu; b Department of Chemistry, Columbia University New York NY 10027 USA wm2256@columbia.edu

## Abstract

Advanced Raman-based techniques, particularly stimulated Raman scattering (SRS) microscopy, have emerged as valuable bioimaging tools. But advances in the development of the chemical toolbox required for detection of biologically relevant species, including metals, has lagged the rapid advances in instrumentation. To date, few responsive probes for dynamic detection of transient and low abundance species have been developed. We describe herein the design and application of CSZin, a small molecule vibrational probe for detection of Zn^2+^ in biological contexts. The probe, a spiropyran design with a strategically positioned nitrile, undergoes a shift in the stretching frequency of the nitrile group and an increase in the SRS intensity in response to metal-induced opening of the spiropyran. We demonstrate detection of Zn^2+^ ions *in vitro*, and the first examples of recognition-based SRS imaging of this metal in live cells. Changes in SRS intensity and ratio reveal changes in basal labile Zn^2+^ pools in normal and tumorigenic RWPE-1 and -2 cells that result from changes in transporter expression and cation uptake related to cancer progression. Proof-of-concept experiments demonstrate that CSZin can also respond to high concentrations of biologically relevant paramagnetic ions, enabling turn-on detection of metals that typically quench fluorescence. The modular nature of the spiropyran-based sensor may enable facile tuning of the selectivity for sensing of other target ions. This work thus paves the way for the development of a new chemical toolbox and detection strategies, complementary to fluorescence, for biological species that are challenging to image.

## Abstract 2

A spiropyran molecular probe with a strategically positioned nitrile undergoes reversible changes in its vibrational spectrum that enable ratiometric imaging of Zn^2+^, and paramagnetic ions, in live cells by Stimulated Raman Scattering microscopy.

## Graphical abstract

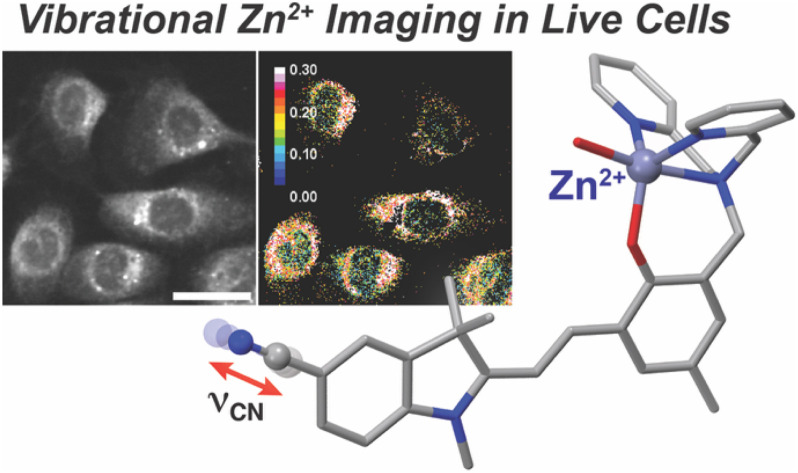

## Introduction

Raman-based microscopy techniques have become an increasingly valuable bioanalytical tool.^1–3^ Unlike fluorescence, most commonly used in bioimaging, Raman scattering is not prone to quenching, thus signal intensity is less susceptible to non-specific interactions in complex biological matrices. Furthermore, Raman scattering can be stimulated with low energy light that results in overall low toxicity to live samples.^4^ A major drawback, however, is the low probability occurrence of the Raman scattering process.^4^ This means that conventional microscopy techniques based on spontaneous Raman are typically too insensitive for imaging applications that require the detection of low concentration species. In this regard, recent advances in non-linear techniques such as Stimulated Raman Scattering (SRS) and Coherent anti-Stokes Raman Spectroscopy (CARS) have largely closed the sensitivity gap and provided enhancement factors that enable single-molecule detection levels without the need for nanostructures or surface enhancement.^5–8^

Among various non-linear Raman techniques, SRS offers linear dependence on concentration and identical spectra to spontaneous Raman, with no interference from the non-resonance background.^2,9^ Furthermore, working in the electronic pre-resonance (epr) regime provides an additional boost in signal intensity that enables epr-SRS detection of suitable chromophores at concentrations as low as nanomolar, competitive with fluorescence.^10^ The linear dependence and sensitivity makes SRS best suited for quantitative studies, thus ideal for bioimaging applications. Perhaps most significantly, the narrow linewidth of typical Raman scattering bands (∼10 cm^−1^), combined with isotopic tuning, makes possible the concurrent detection of a large number of resolvable vibrational modes or ‘colors’.^11^ This feature makes SRS a very attractive alternative for correlative and ‘omics’-type studies that currently require the integration of fluorescence data with other bioanalytical tools across disparate length and time scales.

The advancements in Raman multiphoton microscopy instrumentation, however, have far outpaced the developments in the chemical toolbox necessary to fully realize their potential for biological imaging. Many cellular analytes of low abundance or with small Raman cross-sections, including metal ions and other small reactive species, remain largely out of reach of these techniques. Tagging with chemical moieties that display vibrational frequencies in the silent region for cell imaging (1800–2800 cm^−1^) has allowed background-free tracking of a variety of biomolecules and biological-relevant species using SRS microscopy.^12,13^ For example, alkyne tags have been applied to mark cellular components and structures.^14–16^ And the use of various isotopes on the tags has enabled frequency multiplexing akin to multicolor imaging.

To date, however, there are very few examples of SRS responsive probes whose scattering properties change as a result of interaction with an analyte of interest and thus are able to report on their cellular dynamics. The few examples available include indicators for hydrogen sulfide,^11^ pH,^17,18^ enzymatic activity,^19^ and most recently a Cu^+/2+^-reactive indicator.^20^ At the onset of our work, no SRS indicator had been applied to reversible imaging metal ions *in cellulo*, though metal coordination had been shown to modulate the vibrational features of an alkyne-containing small molecule.^21^ Herein, we describe the design of an indicator for ratiometric detection of biological Zn^2+^ by vibrational spectroscopy, and we demonstrate its application in the SRS mapping of cellular Zn^2+^, including distinct accumulation levels in live normal and tumorigenic prostate cells.

## Results and discussion

### Design and development of a recognition-based, SRS responsive metal indicator

**Fig. 1:**
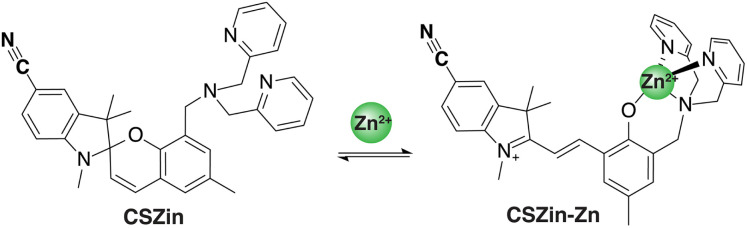
CSZin, a CN-containing sensor for SRS-based detection of Zn^2+^.

**Fig. 2:**
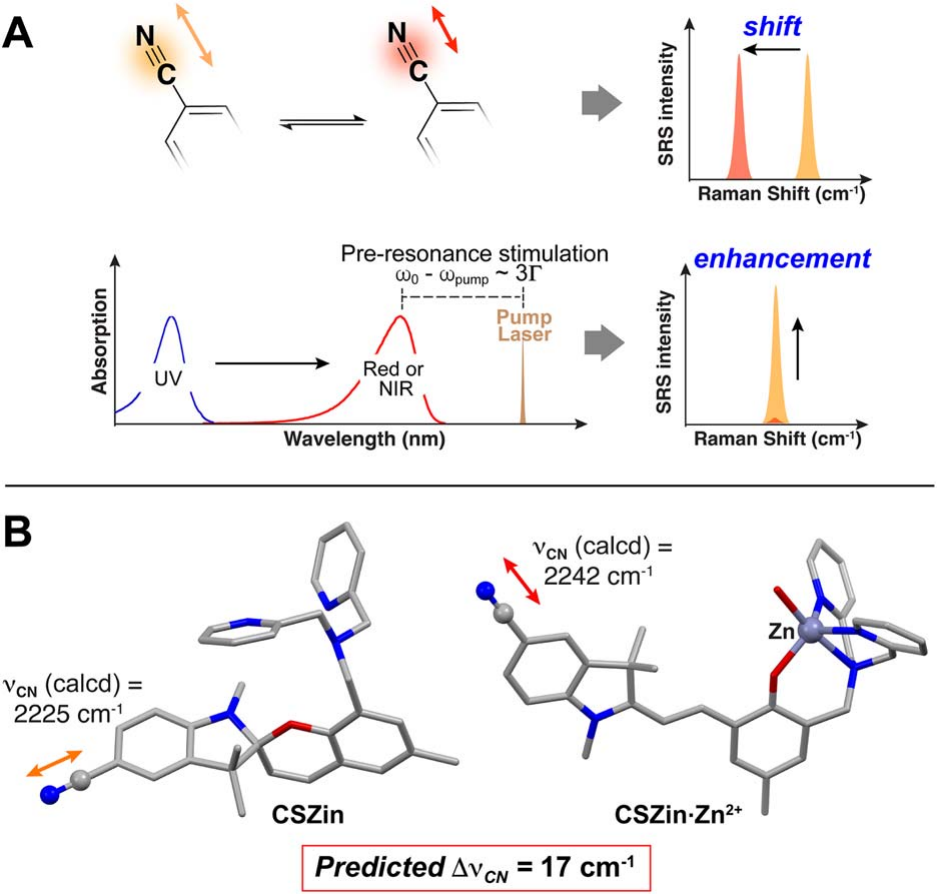
(A) Expected changes in SRS signal in response to metal binding to the sensor. Opening of the spiropyran into a hemicyanine form changes the force constant of the nitrile, modulating its stretching frequency, and it shifts the absorption spectrum to the red, closer to the electronic pre-resonance region thus leading to SRS signal enhancement. (B) Calculated structures of CSZin (left) and CSZin-Zn^2+^ complex (right), with predicted shift in *ν*_CN_ in response to Zn^2+^ binding. Gray = carbon, blue = nitrogen, red = oxygen, purple = zinc. Hydrogen atoms omitted for clarity.

To engineer a metal-responsive sensor, CSZin (Fig. 1), we strategically positioned a nitrile group in the indolenine ring of a spiropyran-based indicator used for fluorescence detection of Zn^2+^ ions in cells.^22^ Binding of the divalent metal cation has been shown to mediate the reversible opening of the benzospiropyran structure to form a hemicyanine species.^22,23^ We reasoned that the change in the electronic structure of the chromophore and development of positive charge in the indolenine ring upon spiropyran opening would result in a change in the force constant of the C≡N moiety, thus producing a change in the stretching frequency that could be used as a dynamic vibrational readout of metal levels (Fig. 2A). Furthermore, we hypothesized that the bathochromic shift in the absorption spectrum of the sensor, which may tail into the red in the Zn^2+^-bound form,^22^ could lead to a metal-induced enhancement of the SRS signal resulting from an epr effect.^10,24^ In this regard, in a setup with pump and Stokes lasers at ∼906 and 1064 nm, respectively, chromophores with absorption maxima between 500 and 700 nm have shown as much as 10^2^–10^4^ enhancement in SRS signal over chromophores that are completely off resonance.^24^

To test the sensitivity of the nitrile vibration to the metal-induced electronic changes in the chromophore, we first investigated the vibrational features of the proposed sensor computationally. Density functional theory calculations were performed on the Zn^2+^-free and-bound forms of CSZin using Gaussian 16, with B3LYP density functional and 6-31g(d,p) basis set for C, H, O and N, and LANL2DZ basis set for zinc.^25^ Solvent (water) was simulated by means of a polarizable continuum model. The initial geometry of the zinc complex was based on the crystal structure of Zn^2+^-bound Zinpyr, a fluorescent sensor with a dipicolylamine metal recognition group that displays a similar coordination environment for the metal center.^26^ An explicit water molecule was used to complete the coordination sphere of the metal. After geometry optimization, Raman frequencies were calculated and corrected.^27^ The frequency of the nitrile stretching was predicted to shift by 17 cm^−1^ upon metal binding and opening of the sensor (Fig. 2B), lending credence to our design hypotheses.

**Scheme 1:**
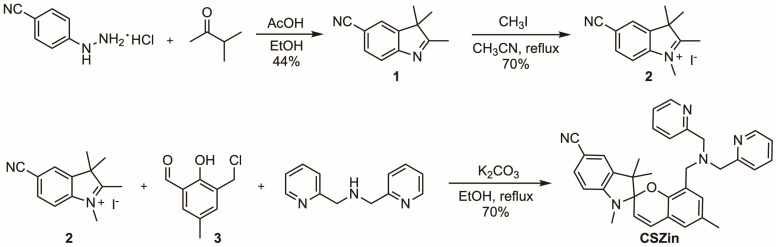
Synthesis of CSZin.

We then proceeded to synthesize CSZin. To this end, a Fisher indole synthesis (Scheme 1) was carried out using 4-hydrazinobenzonitrile and methylbutanone to assemble cyanoindolenine 1. This compound was then alkylated with methyl iodide to afford cyanoindolium salt 2. A one-pot reaction with aldehyde 3 (ref. 22) and dipicolylamine yielded the final sensor, CSZin, purified as a pale yellow solid.

### Vibrational metal detection in solution

**Fig. 3:**
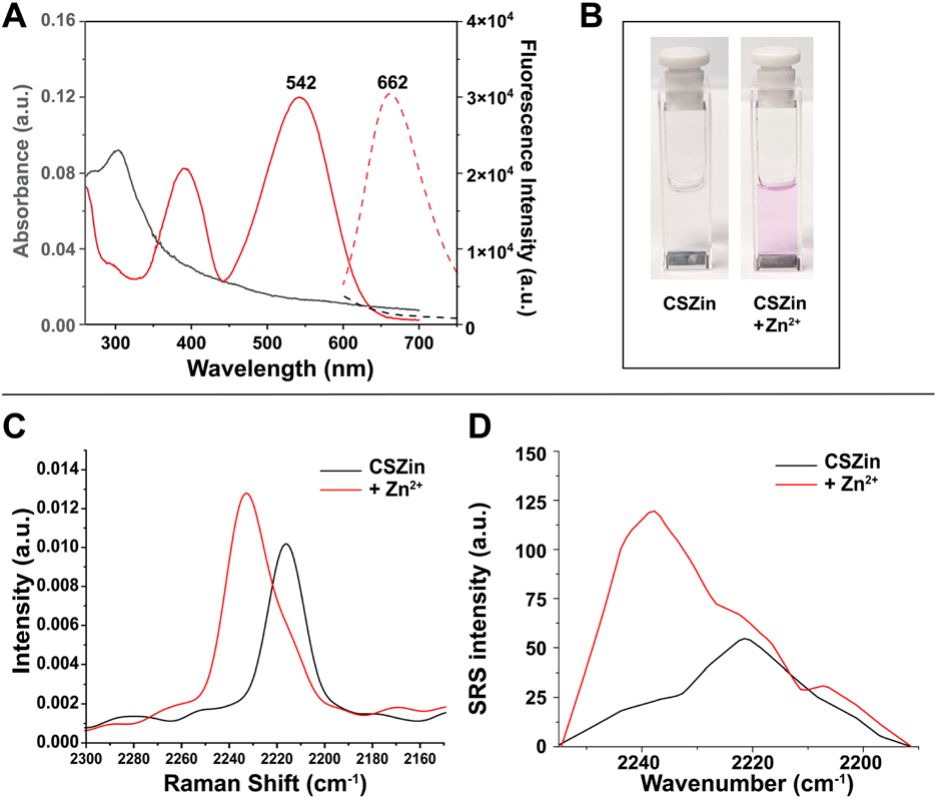
Response of CSZin to Zn^2+^. (A) Representative absorption (solid line) and fluorescence emission (dashed line) spectra of a 5 μM solution of CSZin in aqueous buffer (50 mM PIPES, 100 mM KCl, pH 7.2) before (black) and after addition of ZnCl_2_ (red). (B) Solutions of 5 μM CSZin in aqueous buffer before and after addition of 100 equivalents of ZnCl_2_. (C) Spontaneous Raman spectra, in the nitrile stretching region, of 5 mM solutions of CSZin in 25% DMSO/water containing 5% pluronic, in the absence (black) and presence (red) of 5 equiv. ZnCl_2_, at room temperature. (D) SRS spectra in the nitrile stretching region of 2 mM solutions of CSZin in 25% DMSO/water, in the absence or presence of 25 equiv. of ZnCl_2_, at room temperature.

The metal-binding properties of CSZin were first investigated by UV-vis absorption spectroscopy in aqueous buffer (50 mM PIPES, 100 mM KCl, pH 7.2) containing 5% DMSO. The electronic absorption spectrum of CSZin shows a maximum at 300 nm (Fig. 3A) and no features in the visible range. In the presence of Zn^2+^ ions, the solution turns pink in color (Fig. 3B) and two absorption bands appear at 391 and 542 nm, corresponding to the formation of the hemicyanine species. In its open form, the compound also shows red emission, with a maximum at 662 nm (Fig. 3A). The observed spectral features are slightly red shifted from those of fluorescent indicator SpiroZin1, devoid of the nitrile group.^22^

The kinetics of ring opening at 37 °C were investigated by monitoring the appearance of the absorption band at 545 nm in solutions of the sensor treated with different concentrations of Zn^2+^ (Fig. S1). A control experiment conducted in the absence of Zn^2+^ shows no change in the absorption spectrum over time, indicating no appreciable photochemical opening of the benzospiropyran. When a 5 μM solution of CSZin was incubated with one equivalent of the metal or higher concentration, the absorbance increased and plateaued within minutes. Samples equilibrated accordingly were used in all subsequent experiments.

The apparent dissociation constant of the zinc complex, 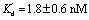, was determined from non-linear fit of the fluorescence intensity as a function of Zn^2+^ concentration in an EGTA-Zn^2+^ buffered solution (Fig. S2). The binding is weaker than that of SpiroZin1,^22^ likely the result of the strong electron withdrawing effect of the nitrile group. Concentrations of labile Zn^2+^ rest at sub-nanomolar levels in most mammalian cells,^28^ but can be substantially higher in tissues such as prostate, pancreas, and the brain; in the latter, they reach an estimated tens of μM in the synaptic cleft and low mM level in zinc-rich vesicles.^29^ Given this wide range, sensors with an equally wide range of affinities are required to track the metal in different samples. The affinity of CSZin is only slightly higher than that of SpiroZin2, another spiropyran-based indicator that has been used successfully for imaging endogenous labile Zn^2+^ in zinc-rich systems.^30,31^

The vibrational response of CSZin to Zn^2+^ was first evaluated experimentally by spontaneous Raman scattering, excited at 785 nm (Fig. 3C and S3). Upon saturation with Zn^2+^, the stretching frequency of the nitrile shifts by 19 cm^−1^, consistent with computational predictions. Encouraged by the results, SRS spectra of the probe were collected in a custom-built SRS microscope (Fig. 3D). A tunable pump beam (855–865 nm) and a fixed Stokes beam (1064 nm) are synchronized timely and overlapped spatially to enable the acceleration of Raman transition by eight orders of magnitude.^10^ For the closed sensor, the nitrile stretches at 2224 cm^−1^. Addition of 0.25 equivalents of zinc chloride results in the appearance of a second well-resolved peak indicating the presence of both metal-free and -bound sensor (Fig. S4). Upon saturation with Zn^2+^, a dominant peak appears at 2241 cm^−1^ and the intensity of the scattering increases 2.8 fold, consistent with a modest pre-resonance electronic enhancement brought about by vibronic coupling with the electronic transitions of the hemicyanine chromophore. The limit of detection for the Zn^2+^-bound species in solution was determined to be 200 μM (Fig. S5).

**Fig. 4:**
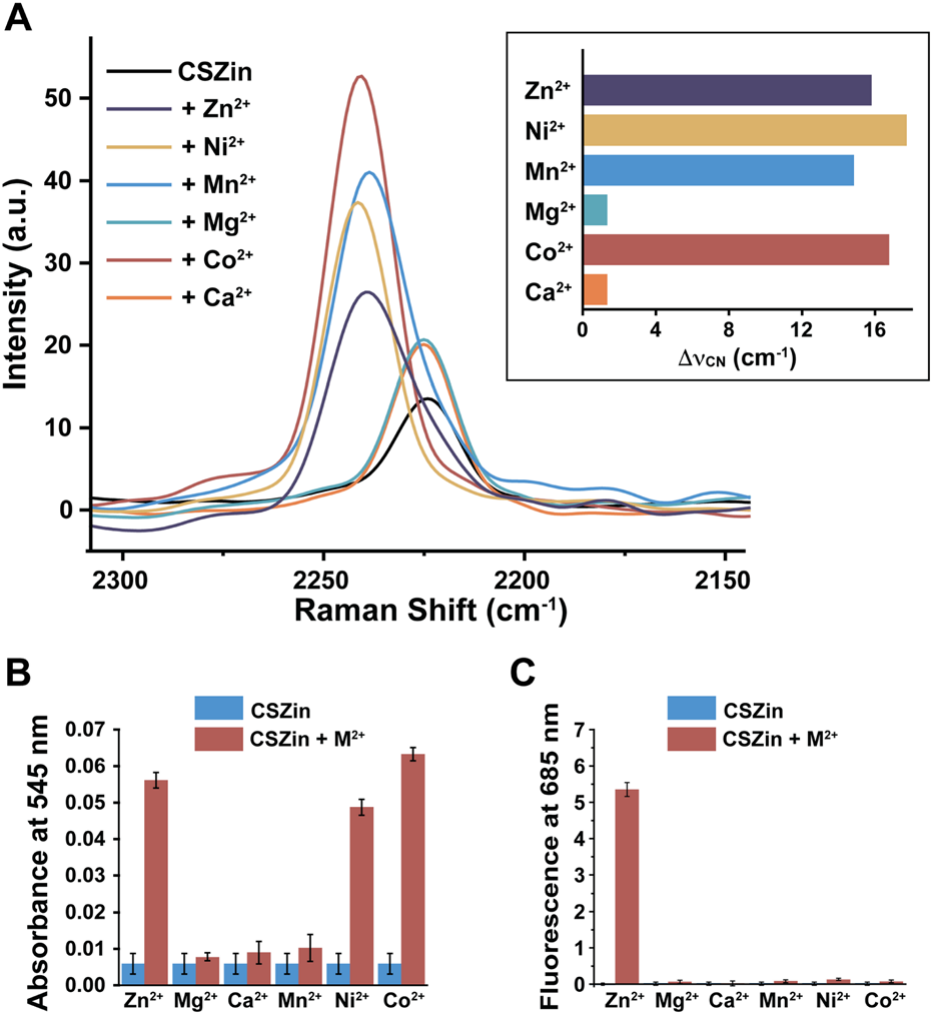
Metal selectivity of CSZin. (A) Raman spectra of 1 mM aqueous solutions of CSZin (1% Pluronic, 10% DMSO) upon treatment with 1 mM divalent metal cations. Inset: nitrile frequency shift relative to unbound sensor. (B) Absorption and (C) fluorescence emission spectra of 5 μM CSZin in aqueous solution (50 mM PIPES buffer, 100 mM KCl, pH 7.0) treated with equimolar concentration of divalent metal cations. For fluorescence, *λ*_exc_ = 545 nm.

The binding of CSZin to other biologically relevant divalent metal cations was then assessed by a combination of UV-visible absorption and fluorescence spectroscopy (Fig. 4 and S6). Spectra were collected for 5 μM solutions of the probe equilibrated with one equivalent of either Zn^2+^, Ca^2+^, Mg^2+^, Mn^2+^, Cu^2+^, Ni^2+^ or Co^2+^, or with equimolar combinations of two metals. The appearance of an absorption band in the visible range reveals the formation of the hemicyanine form with Zn^2+^, Cu^2+^, Ni^2+^, and Co^2+^ but not with the other metal ions at the concentrations tested. Fluorescence, on the other hand, was detected only in the presence of Zn^2+^ (Fig. 4C). In combination, these results indicate that Cu^2+^, Ni^2+^, and Co^2+^ also react with the sensor and induce spiropyran opening, though they quench the fluorescence. The absorption spectrum of solutions containing equimolar mixtures of Zn^2+^ and other metal cations is comparable to that of the solution containing only Zn^2+^, and the fluorescence emission is partially restored in mixtures containing Ni^2+^ and Co^2+^ (Fig. S6). These results suggest that Zn^2+^ partially displaces them under the conditions tested. It should be noted, however, that the biologically relevant concentrations of these metal cations are orders of magnitude lower than those used for this experiment and unlikely to interfere with Zn^2+^ detection.^32^ Only magnesium ions are present at higher concentrations in the cytosol (*ca.* 1 mM),^33^ yet solutions of the probe with 1 mM Mg^2+^ yielded results identical to those with lower Mg^2+^ concentrations, indicating no appreciable binding of the main group cation.

The Raman response to the same cations was then investigated. Spontaneous Raman spectra were acquired for 1 mM aqueous solutions of CSZin in the presence of 1 mM of each metal cation. The solution turns pink and the nitrile stretching shifts to higher frequencies when the sensor is treated with Zn^2+^, Mn^2+^, Ni^2+^, or Co^2+^ (Fig. 4A) indicating complexation and formation of the hemicyanine form. Interestingly, the frequency shift of the nitrile is not the same in response to every metal. Co^2+^ and Ni^2+^ induce slightly larger shifts than Zn^2+^. At the high concentrations used for spontaneous Raman data collection, Mn^2+^ also elicits a vibrational response, with a shift in frequency that is slightly lower than that of Zn^2+^. The absence of the hemicyanine electronic absorption signature at lower concentrations of Mn^2+^, however, suggests that the binding of this metal is quite weak. No large changes were seen with Mg^2+^ or Ca^2+^, consistent with negligible binding and spiropyran opening by these ions. In sum, the probing of Zn^2+^ ions by SRS with CSZin is not expected to be hindered by other biologically relevant divalent metal cations at physiological concentrations.

The results of this study, however, still support the exciting possibility of achieving vibrational readouts for metal ions that quench fluorescence and thus have remained very challenging to image. Given a sensor with suitable affinity for these other metal ions, a ratiometric response would be within reach. In this regard, spiropyran-based switches have been used for the detection of a wide variety of analytes, and the modular nature of the structure is quite amenable to further tuning. The different shifts in frequency observed for CSZin in the presence of various metal ions indicate that the nitrile in our design is not only sensitive to the opening of the spiropyran, but also to the electronics of the metal bound to the hemicyanine. We surmise that changing the position of the nitrile, bringing it in closer proximity to the metal binding site, may intensify this effect and enable a convenient means to distinguish various metals in solution.

### Vibrational imaging of Zn^2+^ in live cells

**Fig. 5:**
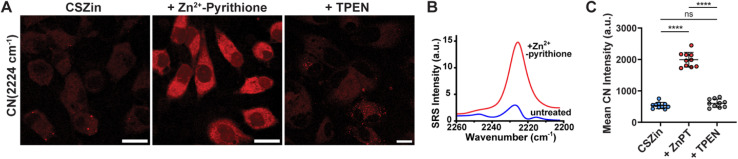
SRS imaging of Zn^2+^ in HeLa cells with vibrational probe CSZin. (A) Nitrile stretching channel, live HeLa cells treated with 20 μM CSZin and 150 μM Zn^2+^-pyrithione or TPEN. Scale bar: 20 μm. (B) SRS spectra of CSZin in HeLa cells untreated with metal (red line) or treated with 150 μM zinc (+pyrithione). (C) Change in SRS intensity of CN bond stretching. ****p ≤ 0.0001; paired *t*-test.

With encouraging results obtained *in vitro*, SRS imaging of Zn^2+^ in live cells was investigated. HeLa cells incubated with 20 μM CSZin for 30 min and either treated with 150 μM Zn (pyrithione)_2_ or non-treated controls were imaged in the nitrile frequency range (Fig. 5). In the Zn^2+^-treated samples, an increase in signal in the CN channel was observed, consistent with the signal enhancement observed in solution due to electronic pre-resonance for the hemicyanine form.^34^ The increase could be abolished by treatment with TPEN, a zinc chelator, thus confirming Zn^2+^ detection by the sensor. To the best of our knowledge, this is the first example of recognition-based SRS imaging of a metal ion in live cells. Cell viability studies demonstrate that CSZin is non-toxic at the concentration used in these experiments, with viability greater than 90% compared to untreated controls (Fig. S7).

The large frequency shift observed upon Zn^2+^ binding *in vitro* appeared diminished *in cellulo*, where only an increase in the average intensity at 2222 cm^−1^, *i.e.* a ‘turn-on’, was observed upon treatment with exogenous Zn^2+^ (Fig. 5B and C). It is possible that the environment encountered by the sensor in the cellular matrix shifts the stretching frequency of the nitrile such that the metal-free and -bound forms are no longer entirely resolvable in our SRS experimental setup. In this regard, we note that the vibrational signatures of nitriles are somewhat sensitive to solvent polarity and the presence of hydrogen bond donors; this feature has been used extensively to study protein structure, though the shifts from changes in local environment are usually modest.^35–37^ To test the effect of local polarity on the vibrational response of the sensor, we acquired spontaneous Raman spectra of the sensor in the absence and presence of Zn^2+^ in various solvents mixtures (Fig. S8). Indeed, the observed Zn^2+^-induced shift in the nitrile stretching frequency was smaller in solvents with lower dielectric constant compared to mixtures with high ratio of water.

Compared to intensiometric imaging, ratiometric response provides more robust detection *in cellulo* as it accounts for variations in sensor concentration in the sample and fluctuations in the illumination source.^38^ Though the inability to resolve the CN stretchings for the metal-free and -bound form precludes us from using them for ratio imaging, the abundance of peaks in the Raman spectrum (Fig. S3) provides useful alternatives for this detection modality. We chose to ratio against the intensity of the double bond band, which changes as the spiropyran structure opens and the fully conjugated hemicyanine form develops. Indeed, the ratio of 2222 cm^−1^ (nitrile) to 1617 cm^−1^ (double bond) signal increases roughly two-fold upon treatment with Zn^2+^, whereas it decreases with subsequent treatment with chelator TPEN (Fig. S9). These results support the feasibility of using a ratio to track Zn^2+^-induced probe switching *in cellulo*.

The fluorescence of the hemicyanine form of CSZin enabled us to conduct a side-by-side comparison of the new SRS imaging method with well-established fluorescence-based imaging of the cation. For this purpose, fluorescence microscopy images were collected of HeLa cells treated with CSZin and Zn^2+^ under similar conditions as our previously described SRS experiments (Fig. S10). As anticipated, addition of Zn^2+^ with pyrithione leads to a significant increase in fluorescence in live HeLa cells loaded with 10 μM of CSZin. Subsequent treatment of the cells with chelator leads to a decrease in the fluorescence signal, consistent with reversible closing of the sensor into its non-emissive form as the metal is removed. Overall, these results indicate that SRS can achieve similar results to more traditional fluorescence-based imaging techniques, while also enabling the visualization of species that have traditionally stumped fluorescence-based detection (*vide infra*). Remarkably, the same perinuclear staining pattern was observed in SRS and fluorescence images of Zn^2+^-treated samples, consistent with fluorescence images of the analogous SpiroZin1.^22^ Fluorescence co-localization analysis with LysoTracker Green DND-26 (Fig. S11) confirmed that CSZin localizes to the lysosome (Pearson coefficient = 0.881), like the parent SpiroZin1 does. These results suggest that addition of the vibrational CN reporter does not have a major impact on the uptake and localization properties of the probe.

### Vibrational imaging of endogenous Zn^2+^ in live prostate cells

**Fig. 6:**
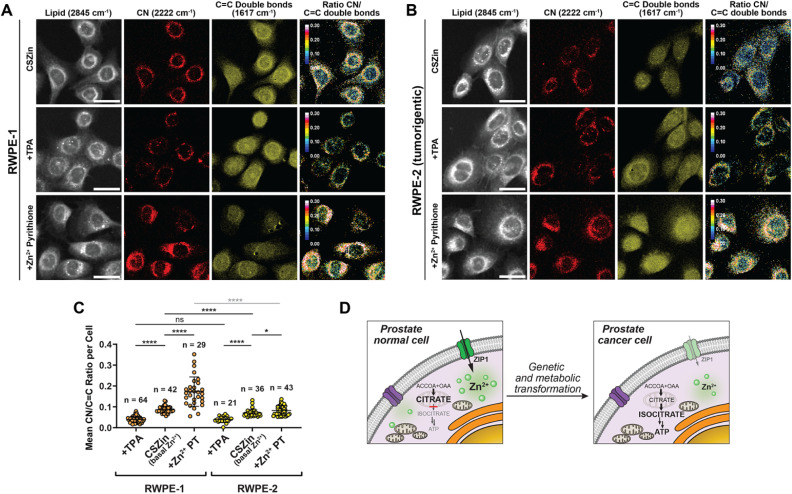
SRS imaging of Zn^2+^ with vibrational probe CSZin in live prostate cells. (A) RWPE-1 cells stained with 25 μM CSZin, grown in 50 μM ZnCl_2_ in the last passage prior to imaging. Top panels: untreated cells. Middle panels: cells incubated with 150 μM TPA. Lower panels: cells incubated with 150 μM Zn(pyrithione)_2_ complex. (B) Tumorigenic RWPE-2 cells stained with 25 μM CSZin, grown in 50 μM ZnCl_2_ in the last passage prior to imaging. Top panels: untreated cells. Middle panels: cells incubated with 150 μM TPA. Lower panels: cells incubated with 150 μM Zn(pyrithione)_2_ complex. Scale bar: 20 μm. (C) Change in CN/C=C double bond ratio. *p ≤ 0.05, ****p ≤ 0.0001, unpaired *t*-test with Welch's correction. Comparisons of samples treated with Zn^2+^ pyrithione should be interpreted with caution, as cell death under excess zinc precluded full equilibration and collection of *R*_max_ in both cell lines. (D) Scheme illustrating changes in cellular zinc accumulation and metabolism in the progression of prostate cancer.

We then sought to investigate the vibrational detection of endogenous levels of Zn^2+^ in a naturally zinc-rich system. Non-tumorigenic prostate cells RWPE-1 and tumorigenic counterparts RWPE-2, previously grown in a Zn^2+^-rich medium, were stained with 25 μM of CSZin for 45 min, washed, and imaged by SRS (Fig. 6 and S12). The CN-to-CC double bond ratio is significantly lower in RWPE-2 than in RWPE-1 cells, indicating that the basal concentration of Zn^2+^ is lower in the former. These observations are consistent with an impaired ability to accumulate Zn^2+^ in the tumorigenic cell line, which arises from downregulation of Zinc importers^39^ that sustains the unique metabolic shift from citrate-producing to citrate-oxidizing that fuels tumor growth in the prostate (Fig. 6B).^40^ To validate the responsiveness of the sensor in these cell lines, and establish the extent of Zn^2+^ binding, the CN-to-CC ratio was determined in cells treated with tris-picolylamine (TPA), a nontoxic Zn^2+^ chelator.^41^ The results show the sensor reaches the same, indistinguishable *R*_min_ value in both cell lines upon zinc depletion, suggesting that the differences observed in untreated samples correspond to true differences in basal labile Zn^2+^ between the two cell lines. Treatment with zinc pyrithione proved toxic for these cells, particularly RWPE-1, which showed extensive blebbing in our experiments. Though an increase in SRS ratio was observed shortly after this treatment, a true *R*_max_ could not be collected due to cell death.

To further validate the results obtained by SRS, fluorescence imaging experiments were conducted on the same cell lines stained with ZnIC,^42^ a well-established iminocoumarin-based fluorescent indicator with a ratiometric response to Zn^2+^ (Fig. S13). The fluorescence ratio of ZnIC showed consistent results with those obtained through vibrational imaging, revealing a lower basal Zn^2+^ content in the tumorigenic RWPE-2 cell. In sum, the results confirm the ability of CSZin to report on changes in endogenous levels of the metal cation and indicate that, with suitable chemical tools, SRS offers a valuable bioimaging alternative for the study of metal homeostasis in health and disease.

### Vibrational imaging of paramagnetic cations in cells

**Fig. 7:**
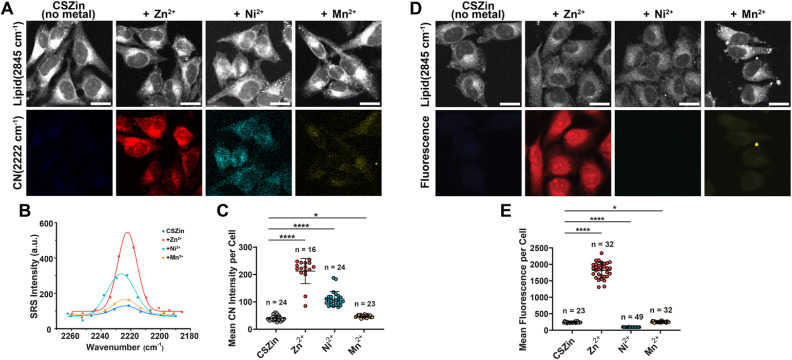
(A) SRS imaging of divalent metal ions with vibrational probe CSZin in fixed HeLa cells. Cells treated with CSZin sensor were subsequently washed and exposed to either Ni^2+^, Mn^2+^, or no metal as control. Same experiment was conducted with Zn^2+^, for comparison. Scale bar: 20 μm. (B) SRS spectra of CSZin collected in images in (A). (C) Change in SRS intensity of CN bond stretching. *p ≤ 0.05, ****p ≤ 0.0001, unpaired *t*-test with Welch's correction. (D) Fluorescence imaging of fixed HeLa cells treated as described in part A. Scale bar 20 μm. (E) Mean fluorescence intensity per cell from images in part D, bottom. *p ≤ 0.05, ****p ≤ 0.0001, unpaired *t*-test with Welch's correction.

Given the results obtained *in vitro*, we conducted proof-of-concept experiments to test the detection of paramagnetic ions *in cellulo* by SRS. For this purpose, HeLa cells were washed with metal-free buffer, fixed, and incubated with 50 μM of CSZin for 40 min. After washing off the excess sensor, cells were incubated with 500 μM of Ni^2+^ or Mn^2+^ for 45 min and imaged by SRS (Fig. 7A). A similar experiment was conducted with Zn^2+^. An increase in signal in the CN channel was observed for all three divalent cations with respect to the untreated control, though the changes were smaller for Ni^2+^ and Mn^2+^ than for Zn^2+^, likely because of their much weaker binding to the probe. For comparison, cells treated in the same fashion were imaged by fluorescence microscopy (Fig. 7D). Fluorescence ‘turn-on’ was observed only upon treatment with Zn^2+^, whereas quenching was observed with Ni^2+^ and Mn^2+^ consistent with results obtained in the cuvette. Overall, though the concentrations of paramagnetic ions used in this experiment are far from physiologically relevant, the results support the feasibility of achieving turn-on vibrational imaging of metals that quench fluorescence and are otherwise difficult to detect. Optimization of the metal recognition motif to furnish stronger and more selective binding of these cations may enable their dynamic imaging by SRS.

## Conclusions

We report herein the development and application of CSZin, a responsive vibrational probe for Zn^2+^ ions designed by strategic incorporation of a nitrile group into a Zn^2+^-sensitive spiropyran switch. The nitrile stretching frequency and the SRS intensity report on the metal-induced formation of the hemicyanine form, thus enabling vibrational detection of the ion *in vitro* and a first example of reversible SRS metal imaging in live cells. The vibrational response of CSZin is suitable for the detection of changes in endogenous levels of the cation in zinc-rich samples, as illustrated in the study of prostate cells. Remarkably, the staining patterns observed in SRS images are comparable with those observed in fluorescence images acquired on similar samples; this unique head-to-head comparison reveals that both imaging techniques afford similar information while the vibrational detection offers some additional advantages.

Although the magnitude of the Zn^2+^-induced frequency shift of the nitrile is smaller *in cellulo* than *in vitro*, precluding its use for ratio imaging, the changes in ratio of the nitrile to double bond signature peaks provides robust ratiometric detection of the cation that facilitate the direct comparison of samples. Overall, compared to the broad, featureless appearance of typical fluorescence emission spectra, the abundance of narrow, well-resolved vibrational features in Raman spectra provide ample opportunities for ratiometric imaging in conceivably most systems. Significantly, these narrow bands make SRS uniquely suited for multiplexing; we envision that nitrile-based vibrational detection of metals could be easily integrated with SRS detection of lipids, metabolites, and other species that would offer unique insight on the interactome of metals in cells.

Proof-of concept imaging experiments conducted with paramagnetic ions support the feasibility of turn-on and ratiometric vibrational detection of metals that typically quench fluorescence and have remained particularly challenging to image. Optimization of the metal recognition group to increase the affinity and selectivity for cations of interest may finally turn this exciting possibility into a reality. The modular synthesis of spiropyran-based switches is very well suited for this endeavor, enabling easy installation of other analyte-recognition groups to diversify the targets of detection. Moreover, the incorporation of other C and N isotopes in the nitrile moiety may enable frequency multiplexing for simultaneous, ‘multicolor’ imaging of various targets using the same base responsive switch.

## Author contributions

E. E. K, S. dM. S., W. M. and D. B. conceived the project. E. E. K., S. dM. S., N. Q., and V. G. V. conducted experiments. W. M. and D. B. secured funding. All authors participated in data analysis and interpretation. All authors contributed to the writing and editing of the final manuscript.

## Conflicts of interest

There are no conflicts to declare.

## Supplementary Material

SC-017-D5SC03442F-s001

## Data Availability

The data supporting this article have been included as part of the supplementary information (SI). Supplementary information: supporting figures, experimental procedures, coordinates of optimized geometries from DFT calculations, and spectroscopic characterization data for new compounds. See DOI: https://doi.org/10.1039/d5sc03442f.
