# Supplementary material for: Imaging intracellular zinc by stimulated Raman scattering microscopy with a small molecule vibrational probe
Source: Chem Sci. 2025 Nov 4;17(1):617–25. doi: 10.1039/d5sc03442f (PMC12599042; doi:10.1039/d5sc03442f)
Supplement: SC-017-D5SC03442F-s001 [file SC-017-D5SC03442F-s001.pdf]

Supplementary Information (SI) for Chemical Science.
This journal is © The Royal Society of Chemistry 2025

Supplementary Information to

Imaging Intracellular Zinc by Stimulated Raman Scattering Microscopy with a Small Molecule Vibrational Probe

Elsy El Khoury[1,2], Symara de Melo Silva[1], Naixin Qian[2], Vinh Vuong[1], Wei Min[2], and Daniela Buccella[1,3]**

[1]Department of Chemistry, New York University, New York, NY 10003, USA
[2]Department of Chemistry, Columbia University, New York, NY 10027, USA
[3]Current address: Department of Chemistry, Emory University, Atlanta, GA 30322, USA

* To whom correspondence should be addressed: daniela.buccella@emory.edu or wm2256@columbia.edu

Table of Contents

1. **Supplemental figures** **2**
2. **Experimental section** **10**
   2.1. Materials and methods 10
   2.2. Synthetic procedures 11
      2.2.1. Synthesis of 2,3,3-trimethyl-3H-indole-5-carbonitrile, 1 11
      2.2.2. Synthesis of 5-cyano-1,2,3,3-tetramethyl-3H-indol-1-ium iodide, 2 12
      2.2.3. Synthesis of CSZin 12
   2.3. Spectroscopic Methods 13
      2.3.1. Determination of metal dissociation constant 14
      2.3.2. Metal selectivity 15
      2.3.3. Determination of SRS limit of detection (LOD) 16
      2.3.4. Study of solvent effects on Raman shift 16
   2.4. Cell culturing protocols 16
   2.5. SRS imaging protocols 16
   2.6. Fluorescence imaging protocols 18
   2.7. Cell viability studies 19
   2.8. Computational methods 20
3. **Spectroscopic data for new compounds** **24**
4. **References** **29**

1. Supplemental figures

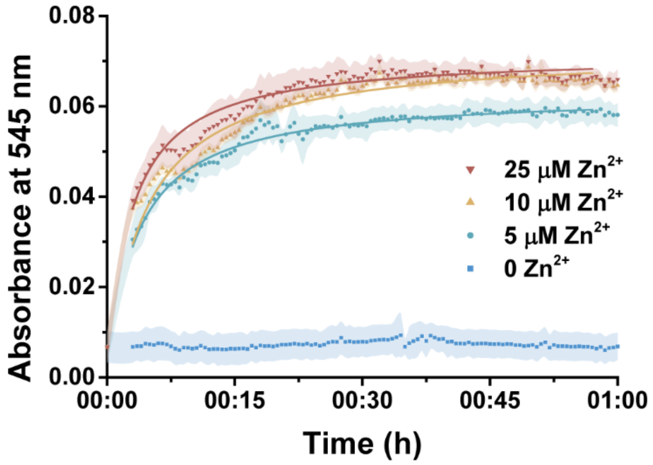

Figure S1. Reaction of 5 μM solution of CSZin with Zn^{2+} in 50 mM PIPES buffer, 100 mM KCl, pH 7.0 at 37 °C. Kinetic curves depicting the change in the absorbance at 545 nm with time, in the presence of various concentrations of $ZnCl_2$.

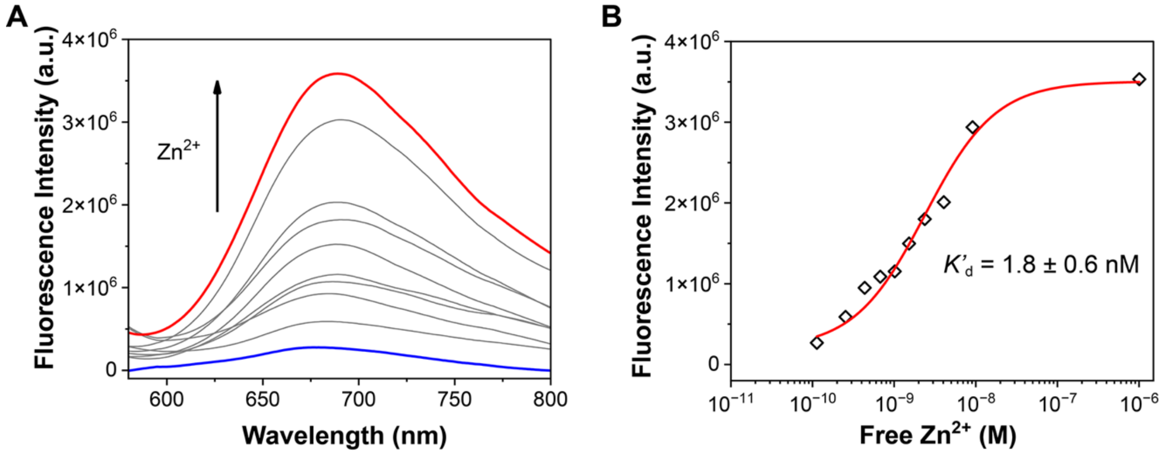

Figure S2. (A) Representative Fluorescence spectra of 5 μM solution of CSZin with increasing concentrations of free Zn^{2+} in aqueous buffer (100 mM HEPES, 100 mM KNO_3, pH 7.0) containing 5% DMSO. Buffered Zn^{2+} solutions were prepared using 1 mM ethylene glycol tetraacetic acid (EGTA) and $ZnCl_2$. λ_{exc}= 545 nm (B) Representative Zn^{2+} binding isotherm from fluorescence data. Apparent equilibrium constant is the average of two independent titrations.

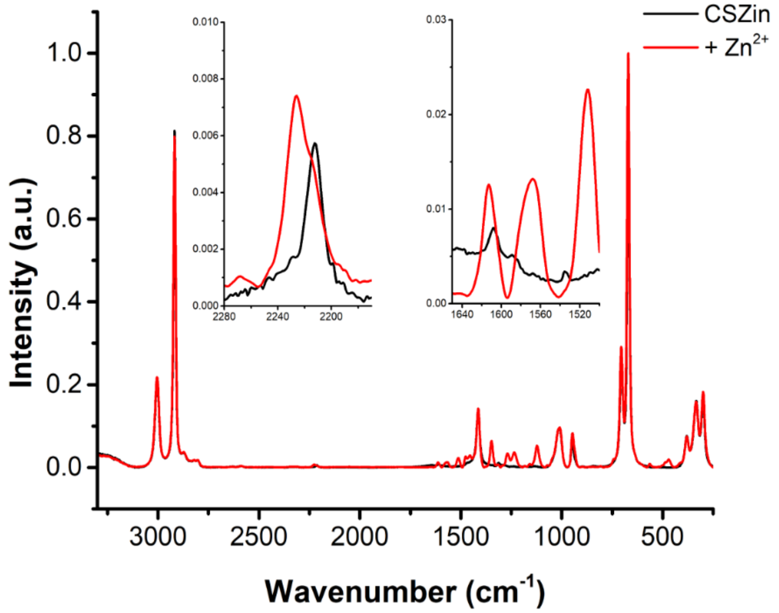

Figure S3. Spontaneous Raman spectrum of CSZin in the metal free (black trace) and metal bound (red trace) forms. 5 mM solutions of CSZin in 25 % DMSO in water containing 5% Pluronic, in the absence (black line) and in the presence (red line) of 25 mM $ZnCl_2$, at room temperature.

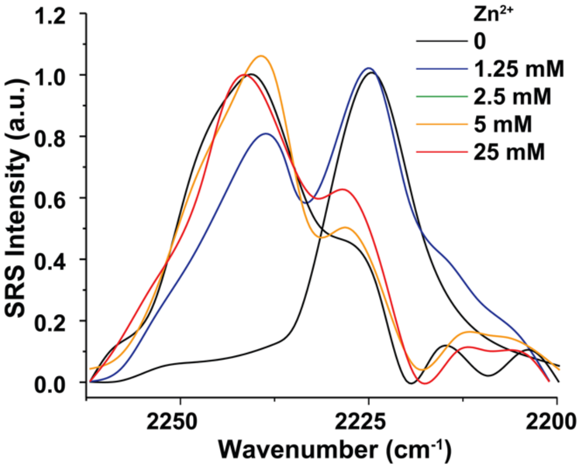

Figure S4. Normalized SRS spectra in the nitrile stretching region of 5 mM solutions of CSZin in DMSO/water (1:1) and 5% Pluronic, in the presence of 0, 1.25 mM, 2.5 mM, 5 mM and 25 mM of $ZnCl_2$, at room temperature.

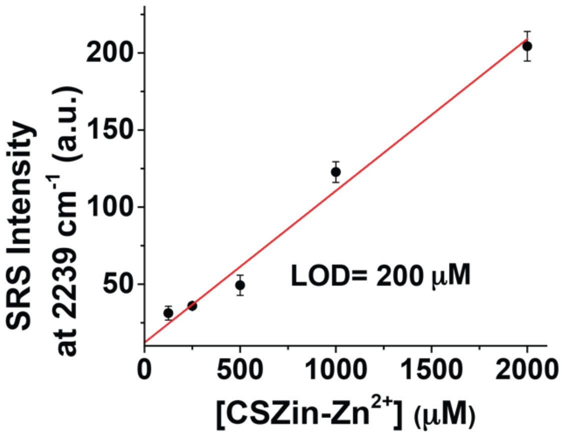

Figure S5. SRS response of zinc-bound CSZin in 20 mM Zn^{2+} aqueous solution containing 20% DMSO.

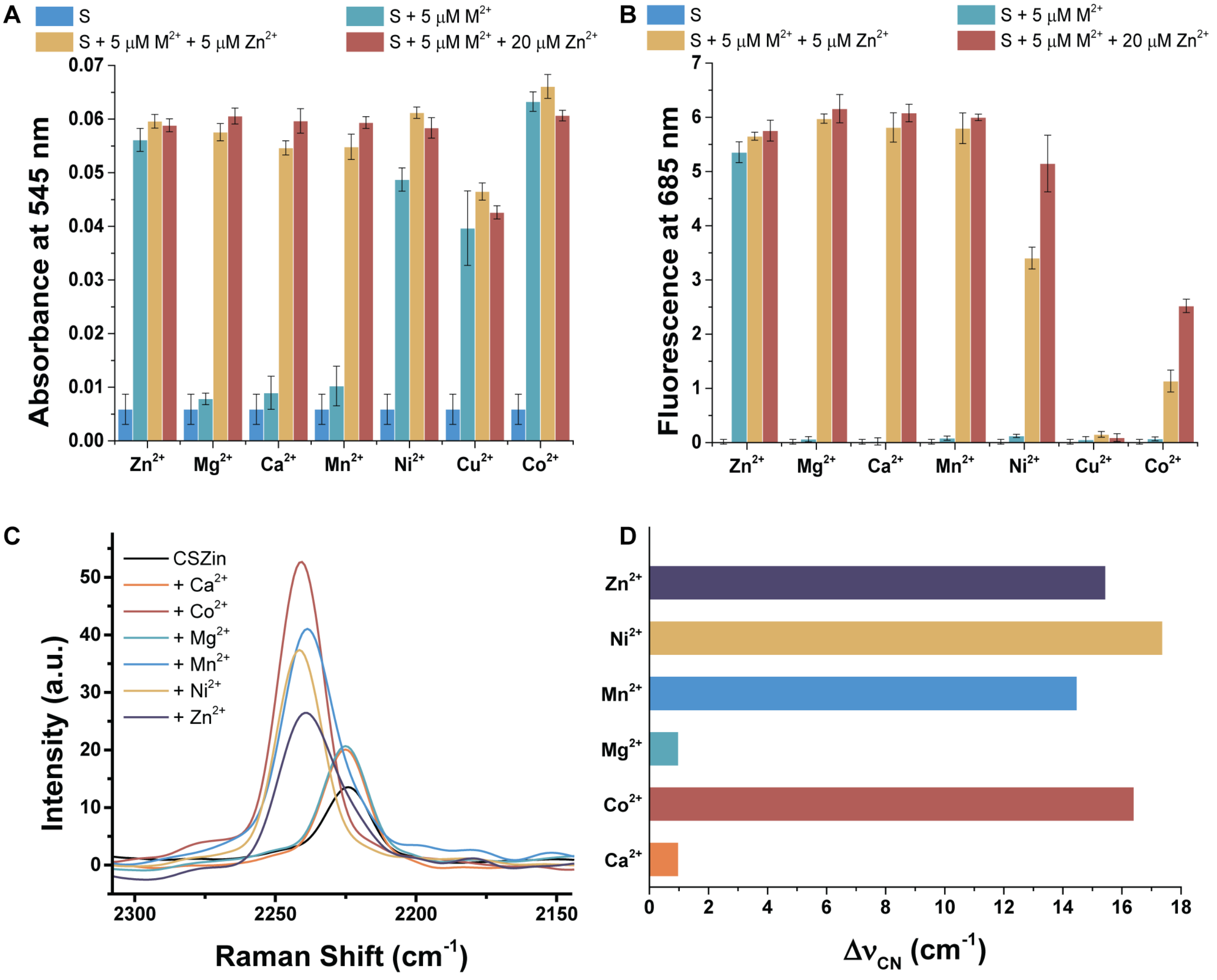

Figure S6. Metal selectivity profile of CSZin in aqueous buffer. (A) UV-Vis absorption intensity, (B) fluorescence emission intensity, and (C) Spontaneous Raman spectra of CSZin in the presence of various metal cations. (D) Nitrile frequency shift in presence of metal, relative to the unbound sensor. Experimental conditions. For UV-Vis absorption and fluorescence: 5 μM sensor and various concentrations of metals in 50 mM PIPES buffer, 100 mM KCl, pH 7.0, 37 °C. Fluorescence excitation at λ_{ex} = 545 nm. For Raman spectroscopy, 1 mM sensor in aqueous buffer containing 1% Pluronic and 10% DMSO, in the absence and in the presence of 1 mM of various divalent metal cations.

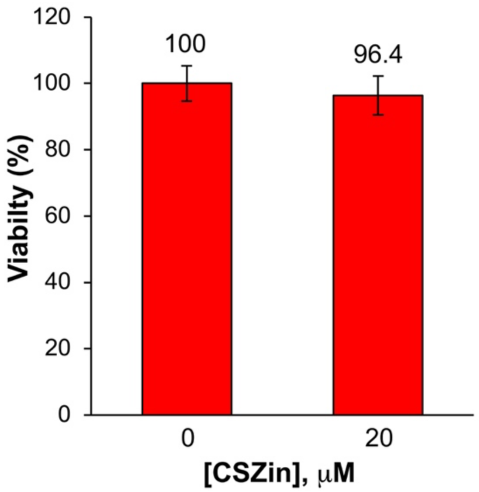

Figure S7. Viability of cells treated with CSZin with respect to vehicle-treated control after 60 min incubation at 37 °C (conditions similar to those used in imaging experiments). Viability measured with *CellTiter Glo*.

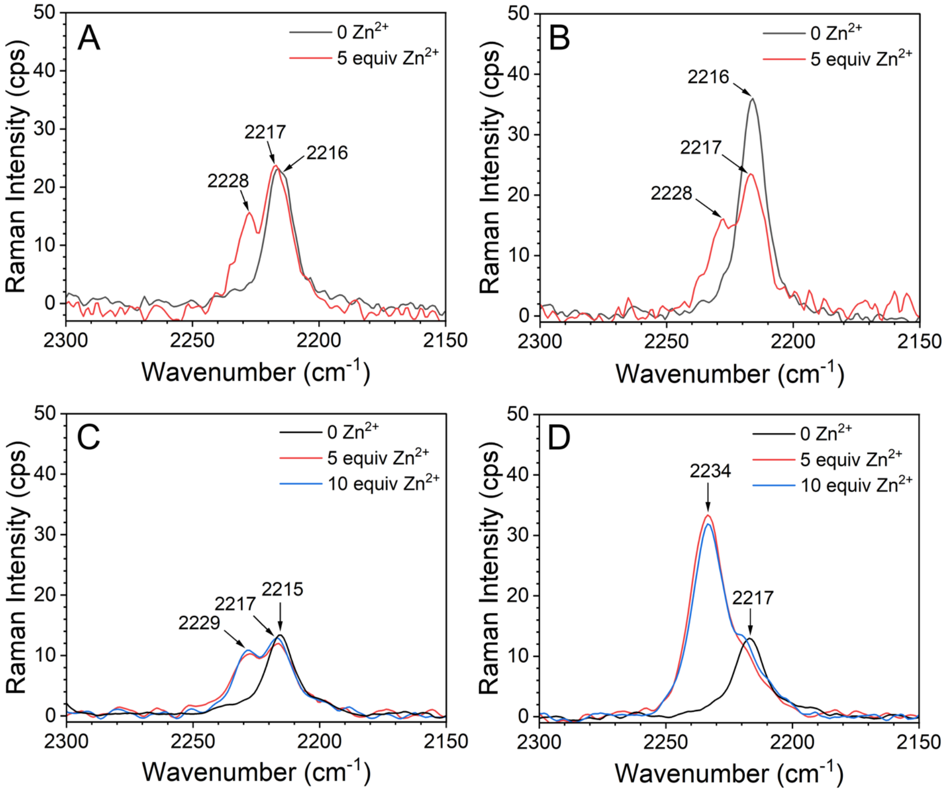

Figure S8. Spontaneous Raman spectra of 5 mM CSZin in the absence (black traces) or in the presence of 5 equivalents of Zn^{2+} (red traces) or 10 equivalents of Zn^{2+} (blue traces) in various solvents mixtures. Measurements conducted in (A) 1:3 DMSO/butanone mixture; (B) 1:3 DMSO/THF mixture; (C) 9:1 DMSO/water mixture; (D) containing 55% DMSO (free) and 30% DMSO (bound) in water. All solutions contain 5% Pluronic.

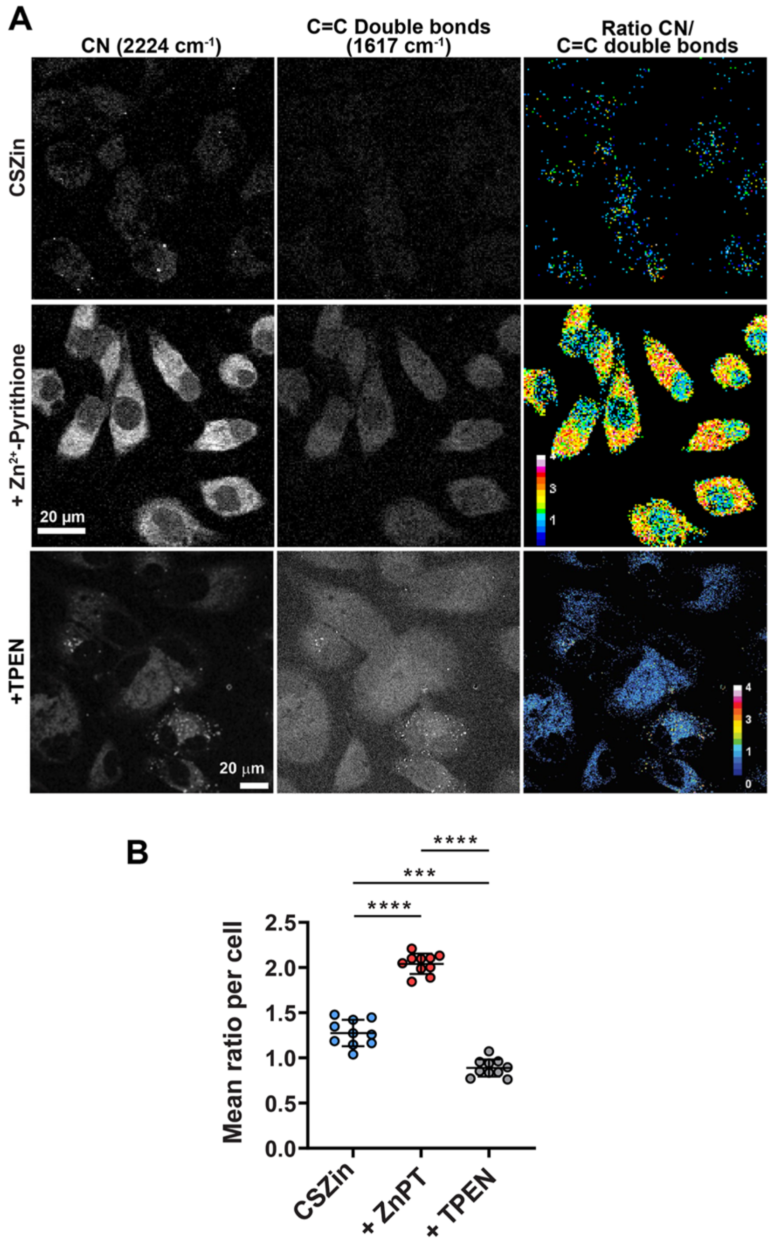

Figure S9. SRS imaging of Zn^{2+} in Hela cells using CSZin. (A) Images in untreated HeLa cells (top) and in HeLa cells treated with 150 µM Zn(pyrithione)$_2$ complex (middle) or with 50 µM TPEN (bottom). Nitrile stretching channel (left), double bond stretching channel (middle), CN/C=C double bond ratio images (right). (B) Change in Raman intensity ratio of CN/C=C double bond when exogenous zinc and TPEN are added to cells. *** $p \leq 0.001$, **** $p \leq 0.0001$ N=10; paired t-test.

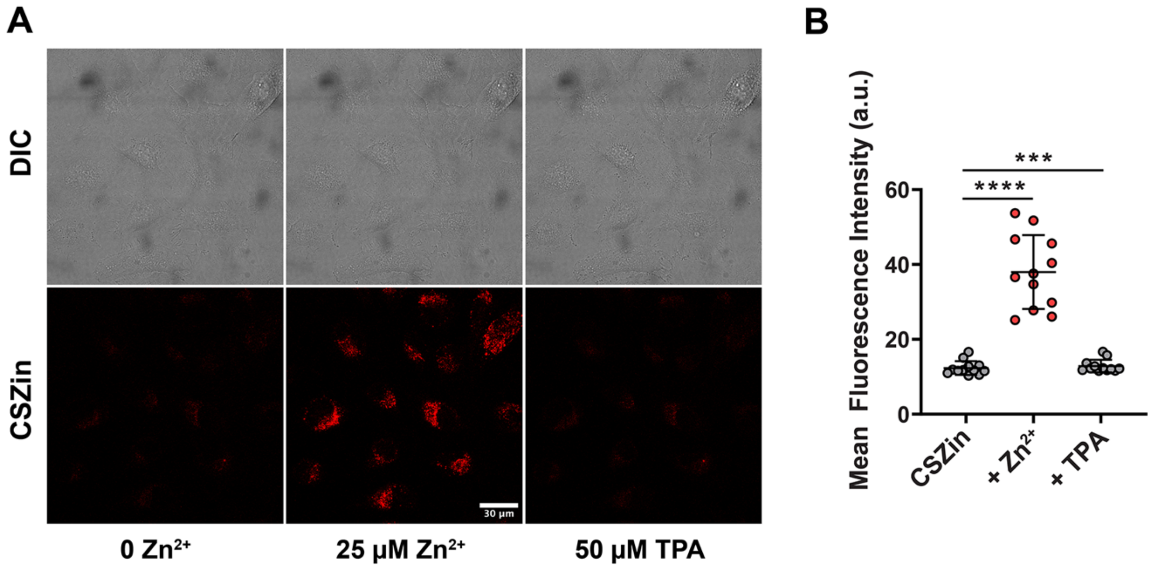

Figure S10. Confocal fluorescence microscopy images of live HeLa cells stained with 10 µM CSZin. (A) Top: Differential interference contrast (DIC) images. Bottom: red channel fluorescence signal from CSZin. Left: no treatment; middle after addition of 25 µM Zn(pyrithione)$_2$ complex; right after addition of 50 µM TPA. (B) Change in fluorescence signal intensity of CSZin per cell. Scale bar: 30 µm. *** $p \leq 0.001$, **** $p \leq 0.0001$, N=12; paired *t*-test.

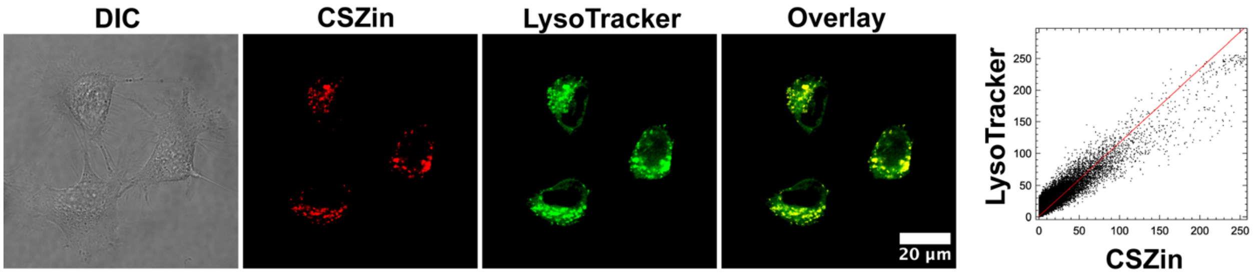

Figure S11. Co-localization analysis of CSZin and a lysosomal marker in HeLa cells. Representative fluorescence images of CSZin (red channel), LysoTracker Green DND-26 (green channel) and corresponding overlap (yellow). Right: Cytofluorogram of pixel intensities from red and green channels. Calculated Pearson's correlation coefficient for the two channels is 0.881.

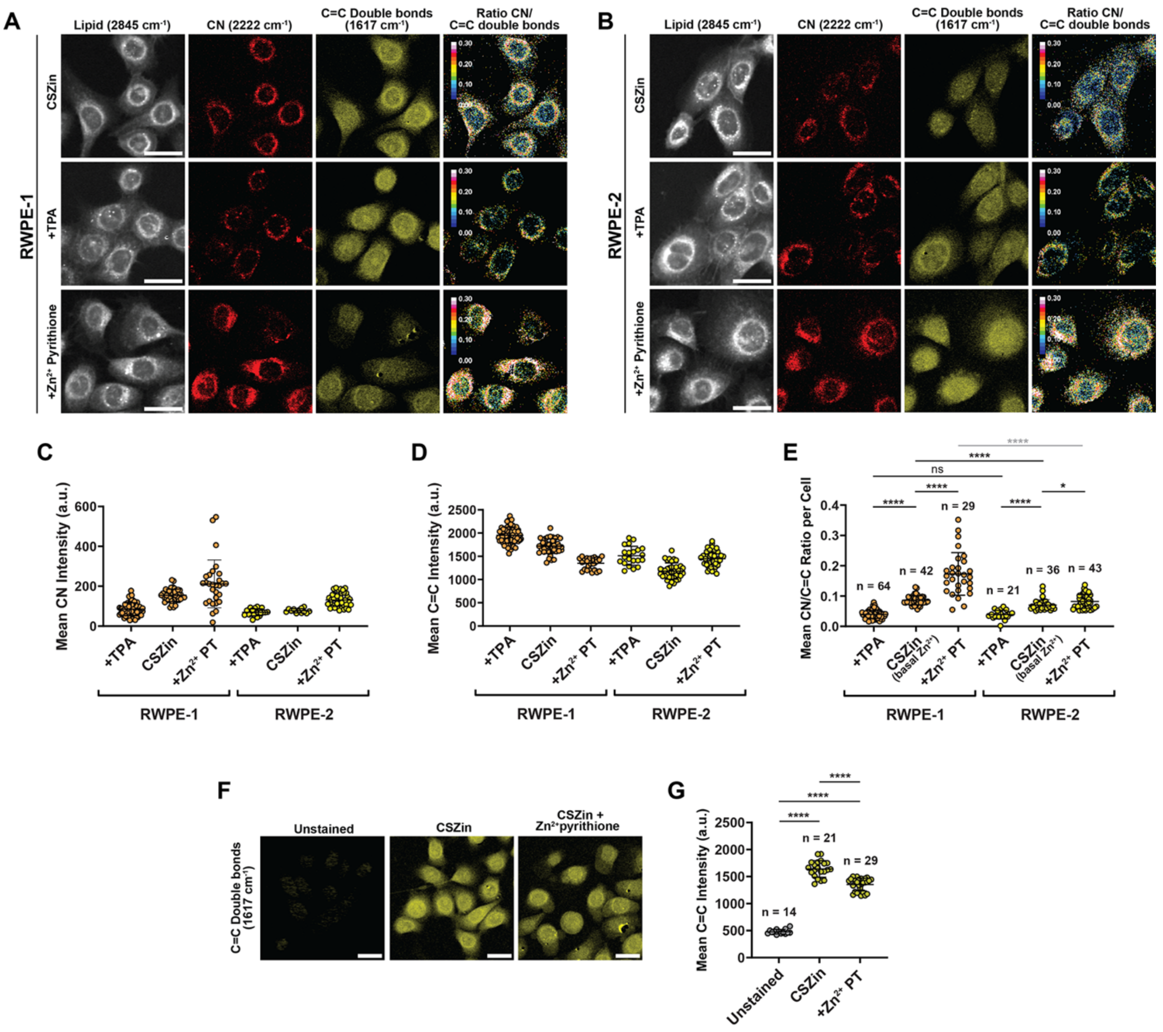

Figure S12. SRS imaging of Zn^{2+} with vibrational probe CSZin in live prostate cells. (A) RWPE-1 cells stained with 25 µM CSZin, grown in 50 µM $ZnCl_2$ in the last passage prior to imaging. Top panels: untreated cells. Middle panels: cells incubated with 150 µM TPA. Lower panels: cells incubated with 150 µM $Zn(pyrithione)_2$ complex. (B) Tumorigenic RWPE-2 cells stained with 25 µM CSZin, grown in 50 µM $ZnCl_2$ in the last passage prior to imaging. Top panels: untreated cells. Middle panels: cells incubated with 150 µM TPA. Lower panels: cells incubated with 150 µM $Zn(pyrithione)_2$ complex. Scale bar: 20 µm. (C) Mean intensity of the CN channel. (D) Mean intensity of the C=C double bond channel. (E) Change in CN/C=C double bond ratio. $*p \leq 0.05$, $****\ p \leq 0.0001$, unpaired *t*-test with Welch's correction. (F) SRS Imaging of live RWPE-1 cells in the C=C region. Left: unstained cells. Middle: cells stained with 25 µM CSZin. Right: cells stained with 25 µM CSZin followed by incubation with 150 µM $Zn(pyrithione)_2$ complex. Scale bar: 20 µm. (G) Change in SRS intensity of C=C, $****\ p \leq 0.0001$, unpaired *t*-test with Welch's correction.

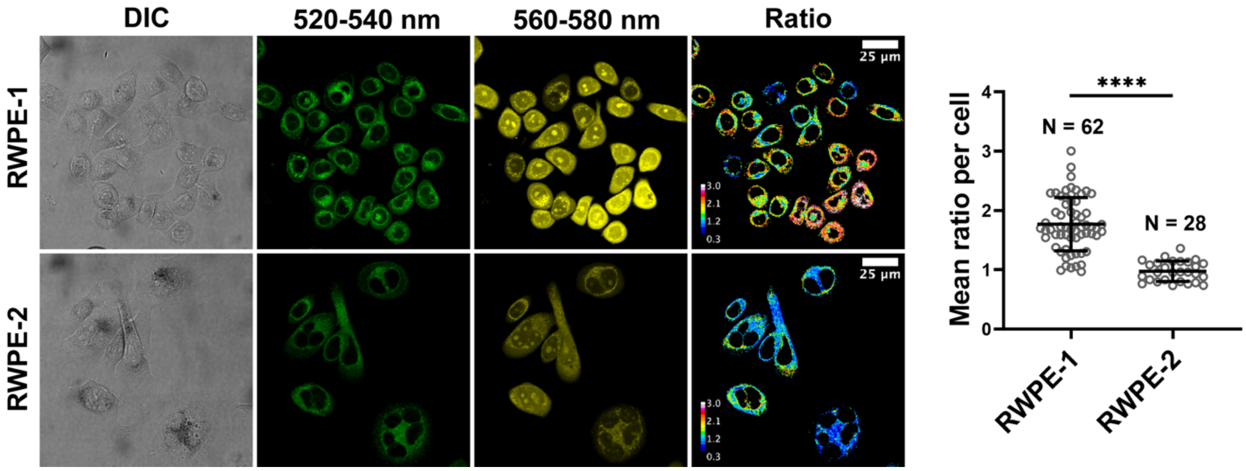

Figure S13. Ratiometric fluorescence imaging of endogenous Zn^{2+} in RWPE-1 and RWPE-2 cells by ZnIC. Cells were stained with 5 µM sensor for 30 minutes at 37°C, washed, and imaged. λ_{exc} = 500 nm. Scale bar: 25 µm. Right: Mean fluorescence ratio $F_{560\text{-}580}/F_{520\text{-}540}$ per cell. **** $p \leq 0.0001$; unpaired *t*-test with Welch's correction.

2. Experimental section

2.1. Materials and methods

4-hydrazinobenzonitrile hydrochloride (98% purity) and bis(pyridin-2-ylmethyl)amine (98% purity) were purchased from TCI America. 3-methylbutan-2-one (98% purity) was purchased from Alfa Aesar. 2,2′-(Piperazine-1,4-diyl)di(ethane-1-sulfonic acid) (PIPES) and 99.999% KCl, were purchased from Sigma-Aldrich. 3-(chloromethyl)-2-hydroxy-5-methylbenzaldehyde was prepared according to the literature.[1] All other reagents and solvents were purchased from commercial sources and used as received. All reactions were carried out under an inert atmosphere, unless otherwise specified. All reactions were carried out using either oven dried or flame dried glassware.

All NMR spectra were acquired on a Bruker Avance III HD-400 NMR spectrometer with cryoprobe. ^1H and ^{13}C NMR chemical shifts are reported in ppm relative to $SiMe_4$ (δ = 0) and were referenced internally with respect to residual protio impurity in the solvent (δ = 7.26 for $CDCl_3$, δ = 3.31 for CD_3OD and δ = 2.05 for $(CD_3)_2CO\text{-}d_6$), or carbon resonance of the solvent (δ = 77.16 for $CDCl_3$, δ = 49.00 for CD_3OD and δ = 29.84 for $(CD_3)_2CO$). High-resolution mass spectrometry

(HRMS) was conducted on an Agilent 6224 Accurate-Mass TOF LC/MS Mass Spectrometer using APCI ionization. Analytical thin layer chromatography (TLC) was performed on SorbTech polyester-backed 200 μm silica gel sheets and SorbTech aluminum-backed 200 μm neutral alumina plates. Flash chromatography separations were conducted using silica gel 40-63 μm (230-400 mesh) and basic alumina 50-200 μm (60 Å, Brockmann Grade I). Reversed-phase HPLC analyses were conducted on an Agilent 1260 system with UV−vis absorption and fluorescence detection, using a Zorbax Eclipse Plus reversed phase column (4.6×50 mm, 1.8 μM particle size) and eluting with a gradient of 25% to 100% acetonitrile/water (+0.1% trifluoroacetic acid) over 14-16 min. Buffered solutions were treated with Chelex-100 resin (Bio-Rad) to remove adventitious metal ions, unless otherwise specified.

2.2. Synthetic procedures

2.2.1. Synthesis of 2,3,3-trimethyl-3H-indole-5-carbonitrile, 1

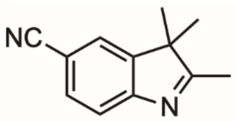

4-hydrazinobenzonitrile hydrochloride (1.57 g, 9.27 mmol) was suspended in absolute ethanol (20 mL). 3-methylbutan-2-one (3.0 mL, 28 mmol) was added, and the reaction was heated at reflux. After 2.5h, acetic acid (1 mL) was added, and the reaction was refluxed for further 2.5h. The solvent was removed *in vacuo* and the reaction was diluted with dichloromethane (100 mL). The organics were washed with saturated $NaHCO_3$ (3 × 50 mL), brine (100 mL), dried over Na_2SO_4 and concentrated *in vacuo* to yield an oily residue. The residue was chromatographed over silica gel (gradient of 15%-20% ethyl acetate in hexanes) to furnish the product as an orange shimmery solid (0.76 g, 44%). R_f = 0.20 (30% ethyl acetate in hexanes, SiO_2). Mp 68-88 °C. ^{1}H NMR (400 MHz, $CDCl_3$) δ: 7.63 (dd, ^4J = 2, ^3J = 8 Hz, 1H), 7.59 (d, ^3J = 8 Hz, 1H), 7.55 (dd, ^4J = 2, ^5J = 1 Hz, 1H), 2.33 (s, 3H), 1.33 (s, 6H). $^{13}C\{^1H\}$ NMR (101 MHz, $CDCl_3$) δ: 192.8, 146.5, 132.9, 125.3, 120.8, 119.5, 108.9, 54.4, 22.9, 15.8. HR-TOF-MS (*m/z*): $[M+H]^+$ calcd for $C_{12}H_{12}N_2$, 185.1073; found 185.1073.

2.2.2. Synthesis of 5-cyano-1,2,3,3-tetramethyl-3H-indol-1-ium iodide, 2

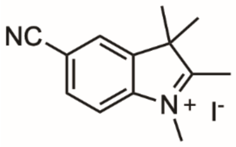

2,3,3-trimethyl-3H-indole-5-carbonitrile (200 mg, 1.10 mmol) was dissolved in anhydrous acetonitrile (2 mL). Methyl iodide (220 µL, 3.30 mmol) was added and the reaction was heated at reflux for 12h. The solvent was removed *in vacuo* and the solid residue was triturated in diethyl ether. Subsequently, the solid was collected by vacuum filtration and further washed with cold acetone/diethyl ether solution (1:1) to furnish the product as a pink powder (246 mg, 70%). Mp 144-196°C (decomp). ^{1}H NMR (400 MHz, CH_3OD) δ: 8.24 (s, 1H), 8.05 (s, 2H), 4.09 (s, 3H), 1.65 (s, 6H).^{13}C{^{1}H} NMR (101 MHz, CH_3OD) δ: 201.6, 146.6, 144.1, 135.1, 128.6, 118.6, 117.6, 114.8, 56.4, 35.8, 22.2. HR-TOF-MS (*m/z*): $[M-I]^+$ calcd for $C_{13}H_5N_2I$, 199.1230; found 199.1233.

2.2.3. Synthesis of CSZin

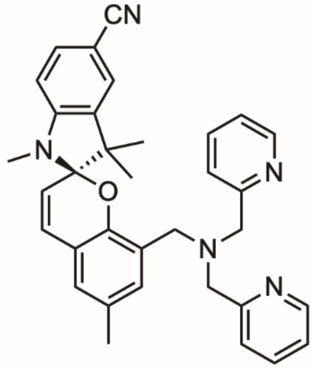

3-(chloromethyl)-2-hydroxy-5-methylbenzaldehyde, **3** (44.7 mg, 0.242 mmol) and potassium carbonate (91.2 mg, 0.648 mmol) were suspended in absolute ethanol (2 mL). Dipicolylamine (100 µL, 0.556 mmol) was added and the reaction was stirred at room temperature for 1h. Then, 5-cyano-1,2,3,3-tetramethyl-3H-indol-1-ium iodide (77.6 mg, 0.238 mmol) was added in one portion and the reaction was heated at reflux for 2h. After colling down to room temperature, the solution was filtered and concentrated *in vacuo* to yield an oil. The oil was dissolved in dichloromethane and washed with water (3 x 5 mL). The organics were further washed with brine (1 x 5 mL), dried over sodium sulfate, and concentrated *in vacuo* to yield a dark orange oil. The oil was chromatographed over basic alumina (gradient of 100% hexanes to 40% ethyl acetate in hexanes) to yield the desired product as a yellow solid (88.5 mg, 70%). R_f = 0.30 (30% ethyl acetate in hexanes, Al_2O_3). ^{1}H NMR (400 MHz, $(CD_3)_2CO$) δ: 8.45 (ddd, $^5J = 1, ^4J = 2, ^3J = 5$ Hz, 2H), 7.69 (td, $^4J = 2$ Hz, $^3J = 8$ Hz, 2H), 7.52 (dd, $^4J = 2$ Hz, $^3J = 8$ Hz, 1H), 7.49 (d, $^3J = 8$ Hz, 2H), 7.44 (d, $^4J = 2$ Hz, 1H), 7.26 (d, $^4J = 2$ Hz, 1H), 7.20 (ddd, $^4J = 1, ^3J = 5, ^3J = 8$ Hz, 2H), 7.01 (d, $^3J = 10$ Hz, 1H), 6.91 (d,

^4J = 2 Hz, 1H), 6.67 (d, ^3J = 8 Hz, 1H), 5.79 (d, ^3J = 10 Hz, 1H), 3.72 (s, 4H), 3.63 (d, ^2J = 14 Hz, 1H), 3.52 (d, ^2J = 14 Hz, 1H), 2.81 (s, 3H), 2.23 (s, 3H), 1.32 (s, 3H), 1.20 (s, 3H). $^{13}C\{^1H\}$ NMR (101 MHz, $(CD_3)_2CO$) δ: 160.6, 152.3, 150.5, 149.7, 138.9, 137.0, 134.2, 132.4, 131.1, 130.0, 127.2, 125.9, 125.3, 123.3, 122.7, 120.9, 119.2, 118.7, 107.7, 104.7, 101.7, 60.6, 52.1, 51.8, 28.9, 26.0, 20.6, 20.3. HR-TOF-MS (*m/z*): $[M+Na]^+$ calcd for $C_{34}H_{33}N_5O$, 550.2583; found 550.2579.

2.3. Spectroscopic Methods

Absorption spectra were obtained using a Cary 100 UV-VIS Spectrophotometer by Agilent Technologies using a 1 cm quartz cuvette. All measurements were conducted at 37.0 ± 0.1 °C maintained by a Quantum Northwest cuvette temperature controller, unless otherwise specified. All aqueous solutions were prepared using deionized water having a resistivity of 18.2 MΩ·cm. All glassware was washed with 10 mM EDTA, rinsed with deionized water and acetone prior to experiments. Stocks of CSZin were prepared in DMSO (25 mM), flash frozen in liquid N_2 and stored at -20 °C. Prior to an experiment, a stock was thawed and then diluted with aqueous buffer to the desired concentration as specified in the figure captions. Data for metal selectivity and binding kinetics were collected on a FlexStation 3 Multi-Mode Microplate Reader from Molecular Devices, conducted in aqueous buffer containing 50 mM PIPES and 100 mM KCl, pH 7.0. Absorption and fluorescence spectra were acquired, using 545 nm to excite the sensor. Spontaneous Raman Spectra were acquired on a Thermo Scientific DXR2 Raman microscope using a 780 nm laser at a 20 mW power for excitation, and a full range grating (50-3300 cm^{-1}) at room temperature. Spectra are the averages of 100 acquisitions at exposure time of 2 seconds through an MPlan 10x/0.25 BD objective and a 50 μm pinhole of 20 μL aliquots, placed in wells on a Hamamatsu microwell slide. SRS solution data and images were obtained with custom microscopes. For initial experiments in live HeLa cells, an integrated laser system (picoEMERALD with custom modifications, Applied Physics & Electronics, Inc.) was used to produce two synchronized laser beams at 80 MHz repetition rate. A fundamental Stokes beam (1064 nm, 6 ps pulse width) was intensity modulated at 8 MHz by an electro-optic modulator with

>90% modulation depth, and a tunable pump beam (720–990 nm, 5–6 ps pulse width) was produced by a built-in optical parametric oscillator. The pump and Stokes beams were spatially and temporally overlapped using two dichroic mirrors and a delay stage inside the laser system and coupled into an inverted laser-scanning microscope (FV1200, Olympus) with optimized near-IR throughput. SRS spectra were acquired by focusing the lasers on the sample through a 60× water objective (UPlanAPO/IR, 1.2 NA, Olympus). The beam sizes of pump and Stokes lasers were adjusted to match the back aperture of the objective. After the sample, both beams were effectively collected by a high-NA oil condenser lens (1.4 NA, Olympus) located above the sample. For imaging of RWPE-1 and RWPE-2 cells, and fixed HeLa cells, an integrated commercial laser source (PicoEmerald S system, Applied Physics & Electronics, Inc.) was utilized to produce both the Pump and Stokes beams for SRS. The wavelength of Stokes beam was fixed as 1031.8 nm, which is modulated at 20 MHz by an electro-optic modulator (EOM). The pump beam is the tunable OPO signal pumped by the second harmonic generation of the IR beam. Both beams had ~2 ps pulse width and 80 MHz repetition rate. The two beams were spatially and temporally synchronized and tightly focused onto the sample with a 25× water objective (XLPlan N, 1.05 NA MP, Olympus). After passing through the sample, both transmitted beams were collected by a 1.4 N.A. oil condenser above the sample. The Stokes beam was filtered off by a high O.D. bandpass filter (890/220 CARS, Chroma Technology), while the Pump beam was detected by a silicon photodiode (S3590-09, Hamamatsu) with a DC voltage of 64 V. The output current was terminated by a 50 Ω terminator and demodulated by a high-frequency lock-in amplifier (HF2LI, Zurich instrument) at 20 MHz frequency. The Pump loss signal at each pixel was digitized and sent to the FV10 analog channel to generate the images.

2.3.1. Determination of metal dissociation constant

Titrations with Zn^{2+} were conducted via fluorescence spectroscopy, using 5 μM of CSZin in aqueous buffer (100 mM HEPES, 100 mM KNO_3, pH 7) containing 5% DMSO, and a buffered zinc system containing 1 mM ethylene glycol tetraacetic acid (EGTA). The concentration of free

Zn^{2+} was varied from 0 to 63 nM by addition of $ZnCl_2$. The solutions containing sensor and the desired amount of Zn^{2+} were equilibrated in the dark for 24 h, although shorter equilibration times might suffice. To determine the apparent dissociation constant, K_d', data were fitted to equation S1

$$F = F_0 + (F_{max} - F_0)\frac{[Zn^{2+}]}{K_d+[Zn^{2+}]} \quad (S1)$$

with F = fluorescence emission at 680 nm at any given point, and F_{max} and F_0 are values of fluorescence emission at 680 for the Zn^{2+}-free and Zn^{2+}-saturated samples, respectively. The reported K_d' is the average of two independent titrations.

2.3.2. Metal selectivity

Metal selectivity experiments by UV-vis absorption and fluorescence spectroscopies were conducted at 37 °C using 5 μM solutions of CSZin in 50 mM PIPES buffer, 100 mM KCl, pH 7.0 in the presence of $ZnCl_2$, $MgCl_2$, $CaCl_2$, $MnCl_2$, $NiCl_2$, $CuCl_2$, and $CoCl_2$, for a final concentration of 5 μM M^{2+} alone, 5 μM M^{2+} in the presence of 5 μM Zn^{2+}, and 5 μM M^{2+} in the presence of 20 μM Zn^{2+}. For magnesium selectivity, an additional concentration of 1 mM Mg^{2+} in the presence of 5 μM Zn^{2+} was used. Once the sensor was added to the metal solution, the absorbance at 545 nm was monitored until equilibrium was reached. Subsequently, the fluorescence intensity at 685 nm was measured at λ_{ex} = 545 nm, and both absorption and emission spectra were collected. Data was extracted using R Studio and plotted using Origin Pro 9.

Metal selectivity experiments by spontaneous Raman were conducted at room temperature using 20 μL aliquots of 1 mM aqueous solutions of CSZin in aqueous buffer containing 1% Pluronic and 10% DMSO, in the presence of 1 mM $ZnCl_2$, $MgCl_2$, $CaCl_2$, $MnCl_2$, $NiCl_2$, and $CoCl_2$. All the raw spectra have undergone a baseline correction, frequency calibration, and smoothing using OMNIC software and OriginPro 9.

2.3.3. Determination of SRS limit of detection (LOD)

A solution of 2 mM CSZin equilibrated with 10 mM $ZnCl_2$ in 20% DMSO/water containing 2% pluronic was serially diluted with the same solvent mixture containing 10 mM $ZnCl_2$, and SRS spectra were collected. A sample containing the same solvent mixture and $ZnCl_2$, in the absence of sensor, was used as blank.

2.3.4. Study of solvent effects on Raman shift

Spontaneous Raman spectra were acquired on samples containing 5 mM CSZin in the absence or presence of 5 or 10 equivalents of Zn^{2+} (from $ZnCl_2$). Solvents tested include mixtures of 1:3 DMSO/Butanone; 1:3 DMSO/THF; 9:1 DMSO/Water; or 3:7 DMSO/Water. All solutions contained 5% Pluronic.

2.4. Cell culturing protocols

Cell lines were purchased from ATCC (Manassas, VA). HeLa cells were cultured in Dulbecco's Modified Eagle Medium (DMEM) supplemented with 10% fetal bovine serum (FBS) at 37 °C in a 5% CO_2 humidified atmosphere. Cells were subcultured every 2-3 days upon reaching 80% confluency, following ATCC guidelines. RWPE-1 and RWPE-2 were cultured in Keratinocyte SFM supplemented with human recombinant epidermal growth factor (rEGF) and bovine pituitary extract (BPE) at 37 °C in a 5% CO_2 humidified atmosphere. Cell were subcultured every 3-4 days upon reaching 80% confluency, following ATCC guidelines.

2.5. SRS imaging protocols

General

SRS images were acquired in a custom-built microscope as described above. Unless otherwise stated, the images are acquired with on-sample pump power of 100 mW and Stokes power of 150 mW. The signal for each channel was normalized to the intensity of pure D_2O that was collected prior to each experiment, to correct for laser intensity changes should they be significant. Images

were collected at a pixel dwell time of 3 μs and the images are averages of 5 frames. Image processing was performed with ImageJ 1.53c.[2] Briefly, background signal from off-resonance images was subtracted from the on-resonance images. A threshold was applied to the resulting background corrected images to remove low-intensity pixels outside the cells and high-intensity pixels caused by highly scattering particles. The cells were then segmented, and regions of interest (ROIs) corresponding to each cell were applied to the rest of the processing. The average intensity per cell was recorded. To obtain the ratio of nitrile to double bond signals, the nitrile channel image was divided by the double bond image, and the average ratio per cell was calculated from the ROIs.

SRS imaging of Zn^{2+} in live HeLa cells

Cells were seeded on cover slips in 4 well plates 24 h prior to imaging. At the time of imaging, cells were washed with phosphate buffered saline (PBS) and incubated at 37 °C for 30 minutes in phenol red-free and sodium pyruvate-free DMEM containing 25 μM CSZin. The dye-containing medium was then removed, the cells were washed twice with PBS and bathed in 0.5 mL of the imaging medium (Phenol red free DMEM). An imaging spacer was placed between the cover slip and a glass slide creating a chamber where the cells remained bathed in imaging medium. For imaging in the presence of Zn^{2+}, a 150 μM solution of zinc-pyrithione 1:2 complex (150 μM in Zn^{2+}) in the imaging medium was used in the last step. For imaging under Zn^{2+}-depleted conditions, a 50 μM solution of TPEN in the imaging medium was used in the last step. Images were acquired after an incubation of at least 15 minutes.

SRS imaging of Zn^{2+} in prostate cells, RWPE-1 and RWPE-2

RWPE-1 or RWPE-2 cells were plated three days before imaging and grown on a coverslip in keratinocyte SFM supplemented with 50 μM zinc chloride. At the time of imaging, cells were washed twice with DPBS and incubated in growth media containing 25 μM CSZin for 45 minutes. After two additional DPBS washes, cells were mounted on a glass slide and imaging was performed in phenol red-free DMEM-F12K media as indicated above.

SRS imaging of paramagnetic ions in fixed HeLa cells

HeLa cells were plated and grown on coverslips for two days before imaging. On the day of imaging, cells were washed twice with DPBS without Ca^{2+} and Mg^{2+} ions (buffer used in subsequent steps), then fixed with 4% PFA at room temperature for 20 minutes, washed three times with DPBS and incubated in the same buffer containing 50 µM of CSZin for 40 minutes at 37 °C. Finally, after two washes with DPBS, the cells were incubated in the presence of 500 µM of Mn^{2+} or Ni^{2+} in the same buffer at 37 °C for 45 minutes. For comparison, an experiment with Zn^{2+} was conducted under the same conditions. Cells were mounted on a glass slide and imaged in the corresponding metal-containing buffer solution.

2.6. Fluorescence imaging protocols

General

Fluorescence imaging was performed on a Leica TCS SP8 X laser confocal microscope equipped with a 63× glycerol immersion objective (HC PL APO CS2 63×/1.30 GLYC), GaAsP hybrid detectors, a tunable white-light laser source, a Leica DFC310 FX digital color camera, and a Okolab stage-top environmental chamber. The microscope was operated with Leica LAS AF software. Image processing was performed with FIJI. For imaging CSZin, images were acquired with excitation at 545 nm and emission collected 600−680 nm. After subtraction of the background, ROIs were defined for each cell and average fluorescence intensity recorded. For imaging ZnIC, fluorescence was excited at 500 nm, and emission was collected at 520–540 nm (Zn^{2+}-free form) or 560–580 nm (Zn^{2+}-bound form). Laser power (10%), detector gain (green channel 50; yellow channel 10), and other acquisition parameters were kept constant to enable direct comparison of fluorescence ratios between experiments. After subtraction of the background, ROIs were defined for each cell used for the rest of the processing. For ratio images, corrected images for the Zn^{2+}-bound channel were divided by corresponding corrected images for the Zn^{2+}-free channel, and the average ratio per cell was calculated from the ROIs. LysoTracker

Green DND-26 fluorescence was excited at 480 nm and collected at 520-540 nm. Colocalization analysis was performed using the JaCoP plugin in Fiji. Background subtraction and intensity thresholding were applied to each channel prior to analysis.

Fluorescence imaging of CSZin in HeLa cells

One day prior to imaging, 0.25-0.3x10^6 HeLa cells in complete medium were seeded in 35 mm glass bottom cell culture dishes (MatTek, Part no. P35GC-1.5-14-C). At the time of imaging, cells were incubated at 37 ºC for 45 minutes with CSZin (10 μM) in DMEM containing 10% FBS and phenol red. Following incubation, the cells were washed with PBS (2×2 mL) and bathed in imaging media (Live Cell Imaging Solution, Molecular Probes, 2 mL) for image acquisition. Cells on the microscope stage were treated with 25 μM of Zn(pyrithione)$_2$ followed by 50 μM TPA.

Organelle localization analysis of CSZin

Two days before imaging, 0.25-0.3x10^6 HeLa cells in complete medium were seeded in 35 mm glass bottom cell culture dishes. Prior to imaging, cells were washed with DPBS (1×2 mL) and incubated with 20 μM CSZin in FBS-free DMEM for 30 minutes at 37 °C. Cells were washed with DPBS (2×2 mL) and further incubated with LysoTracker Green DND-26 (Invitrogen cat. no. L7526) for 10 minutes at 37 °C. Cells were washed with DPBS (2×2 mL) and bathed in Live Cell Imaging Solution (2 mL) containing 10 μM of Zn(pyrithione)$_2$ for image acquisition.

Fluorescence imaging of Zn^{2+} in prostate cells, RWPE-1 and RWPE-, using ZnIC[3]

Cells were seeded two days before imaging in complete Keratinocyte SFM medium supplemented with 50 μM $ZnCl_2$. At the time of imaging, cells were washed with DPBS (1×2 mL) and incubated with 5 μM ZnIC in complete medium for 30 min at 37 °C. After incubation, cells were washed twice with DPBS (2×2 mL) and bathed in Live Cell Imaging Solution (2 mL) for image acquisition.

2.7. Cell viability studies

The viability of cells treated with CSZin complex under the conditions used for imaging was evaluated with the *CellTiter Glo* luminescent assay (Promega, Catalog No. G7570). HeLa cells

(6,000 cells per well) were seeded in a white 96-well flat-bottomed plate. The plate was incubated at 37°C in a humidified 5% CO_2 atmosphere for 24 h prior to treatment. Cells were washed with DPBS (1×200 µL) and bathed in a solution of 20 µM CSZin in complete growth medium (100 µL, DMEM + 10% FBS). The plate was incubated at 37°C in a humidified 5% CO_2 atmosphere for 60 minutes. Cells were then treated with 50 µL of CTG reagent and incubated at room temperature for 30 minutes, followed by measurement of luminescence at 560 nm. Experiments were conducted in triplicate for each concentration.

2.8. Computational methods

Density functional theory (DFT) calculations were performed using Gaussian 16 revision A03. Geometry optimizations and Raman frequency calculations in the ground state were run with B3LYP hybrid functional and 6-31G(d,p) basis set for C, H, N and O and LANL2DZ for zinc. Water was used as solvent in a polarizable continuum model (PCM). All frequencies were corrected by a factor of 0.964.[4]

Coordinates of geometry optimized CSZin

N	-1.48643274	1.70537147	-0.86716031
N	1.52884510	2.25587208	-0.79420543
N	-1.79354733	2.38159827	2.07158407
O	-0.30626692	-1.22844849	-0.28222695
C	-3.80464634	-0.86066148	-1.20985758
C	-4.42475922	-1.93131010	-0.54910257
C	1.16640042	2.25057618	-3.17110626
C	-3.62601540	-2.74260656	0.25763190
C	-2.43372686	-0.59422566	-1.10860337
C	-1.65464493	-1.46814268	-0.33371181
C	0.71055013	2.39029789	-1.85536085
C	-2.67182077	2.26190044	-0.20014163
C	-1.82619756	0.60876328	-1.79595379
C	2.81831628	1.98136538	-1.02754962
C	-0.72922467	2.76077870	-1.55491963
C	-2.26163055	5.13443301	2.27652846
C	-2.32533638	3.06474129	1.03952779
C	-2.57537819	4.43985561	1.10775170

C	3.36261713	1.83001684	-2.30506930
C	-1.70683551	4.43108414	3.34272529
C	-2.24598841	-2.52636665	0.37500775
C	2.51321164	1.96748232	-3.40036854
C	-1.49521451	3.05949495	3.18641372
H	0.47529055	2.37005626	-3.99954337
H	-3.27846663	2.88350516	-0.88385293
H	-3.29929810	1.42373805	0.11280583
H	-2.51921559	0.95537906	-2.58456546
H	-0.89736501	0.31420680	-2.29309393
H	3.45386353	1.88187017	-0.14935003
H	-0.70743673	3.63277047	-0.89440310
H	-1.23063868	3.07645784	-2.48811914
H	-2.44630285	6.20188264	2.34956125
H	-3.00989886	4.95368121	0.25602635
H	4.41850471	1.61435074	-2.42948838
H	-1.44568009	4.92381415	4.27324741
H	2.88902397	1.85835257	-4.41316053
H	-1.06935659	2.47734458	4.00167660
C	-5.90307429	-2.19619223	-0.71376910
H	-6.10830632	-2.75555587	-1.63495384
H	-6.29648688	-2.78508458	0.11960496
H	-6.47296564	-1.26359176	-0.76985508
H	-4.40937744	-0.19802251	-1.82503156
H	-4.07224103	-3.56383749	0.81373251
C	-1.38478005	-3.31970532	1.23747936
H	-1.84088374	-4.07590397	1.87100178
C	-0.06261419	-3.11053076	1.26343710
H	0.59053981	-3.68438510	1.91352849
C	0.62169341	-2.08518440	0.39540059
C	2.76769750	-1.68862185	1.22066359
C	2.94914476	-2.62705329	0.17766030
C	1.79111508	-1.84401558	-1.88991069
C	3.81964182	-1.36274258	2.08915646
C	4.17067980	-3.23232465	-0.01208504
H	0.86994244	-1.86770230	-2.47773022
H	2.00887395	-0.80530406	-1.63394824
H	2.60735139	-2.22394292	-2.51178039
C	5.05330002	-1.97826545	1.89421447
H	3.68443146	-0.65872982	2.90199044
C	5.24379161	-2.91195366	0.85902811
H	4.32307386	-3.95161334	-0.81052616
H	5.87898900	-1.74420725	2.55803355
C	6.51236675	-3.54089375	0.68965538
N	1.48233280	-1.20665230	1.21701756
C	0.91886272	-0.40684607	2.29090832

H	1.60888564	0.40420250	2.53577069
H	-0.01335499	0.04525522	1.94861100
H	0.72663319	-0.99135049	3.20087552
C	1.66290915	-2.72778741	-0.62903997
C	1.29925774	-4.15929748	-1.04708229
H	0.31864924	-4.18354464	-1.53275022
H	2.03302951	-4.53263186	-1.76828224
H	1.27882492	-4.84848425	-0.20000608
N	7.54937906	-4.05597987	0.54769708

Coordinates of geometry optimized CSZin Zn^{2+} complex

Zn	2.20512998	-0.91680773	0.39644811
O	0.70168932	-0.00745544	-0.58594870
N	3.62659842	0.75391653	-0.04296552
N	3.59197778	-1.88111343	-0.95610184
N	2.96720337	-0.40736516	2.37745594
N	-5.52694653	0.96044897	-0.41898241
C	5.64643126	-1.58599118	-2.14474657
C	5.43344234	-2.78401213	-2.82491154
C	4.90685731	0.08972314	-0.37765302
C	4.70456039	-1.16314184	-1.20755066
C	4.28033537	-3.52325070	-2.55894552
C	4.30530729	1.13822116	3.61680156
C	3.75194705	1.57559138	1.17485131
C	4.15199328	0.36440989	4.76519086
C	3.68936759	0.72927271	2.43342590
C	3.38065997	-3.03249942	-1.61956784
C	3.10964226	1.51583252	-1.21810949
C	3.39216444	-0.80470997	4.70277451
C	2.82053600	-1.15613689	3.48628250
C	1.73158476	2.11045334	-1.04099682
C	1.54803471	3.46817800	-1.23390837
C	0.59166480	1.27045943	-0.78657510
C	0.27563194	4.09996804	-1.21542177
C	0.16718400	5.58934891	-1.43310605
C	-0.71559102	1.89806068	-0.78893308
C	-0.82607807	3.30166897	-1.00520313
C	-1.83583661	1.05573940	-0.57433713
C	-3.17402659	1.41933583	-0.58115027
C	-3.50382703	-1.25568005	1.29155841
C	-3.59585276	-1.78775110	-1.21240358
C	-4.21619962	-0.96598243	-0.05381652
C	-4.24207709	0.53731652	-0.36900211

C	-5.70109120	-1.26310147	0.06180040
C	-5.93368672	2.33677197	-0.70536334
C	-6.35945554	-2.44766711	0.33433644
C	-6.42965529	-0.08914418	-0.16237853
C	-7.76999718	-2.43560265	0.38110192
C	-7.82239499	-0.05335085	-0.11969925
C	-8.48767244	-1.24602601	0.15571162
H	6.52815290	-0.98376039	-2.33455679
H	6.15450032	-3.13170680	-3.55748896
H	5.58944332	0.77876766	-0.89117940
H	5.39158977	-0.20021080	0.56139296
H	4.66903173	2.17846058	1.17020883
H	4.89217392	2.05017954	3.63121234
H	4.07474026	-4.45609674	-3.07085341
H	4.62256970	0.66752322	5.69479563
H	3.81833087	2.31236764	-1.48245085
H	3.09575884	0.80781628	-2.05438473
H	2.90946670	2.27519073	1.19101038
H	2.42466693	4.08408001	-1.42626888
H	3.24856865	-1.43533896	5.57232422
H	2.46298887	-3.55916008	-1.38067955
H	2.22541696	-2.05703892	3.38201241
H	0.71228998	6.14570010	-0.66171621
H	0.59304984	5.88298244	-2.39951745
H	-0.87506849	5.91779103	-1.41081024
H	-1.57159909	0.01962508	-0.40056782
H	-1.81098629	3.75893475	-0.99766976
H	-2.43831498	-1.02305814	1.23883354
H	-2.52924782	-1.58473366	-1.32463224
H	-3.43034346	2.45490499	-0.77111774
H	-3.60858717	-2.31645870	1.53471921
H	-3.94435277	-0.67228372	2.10411325
H	-3.71684747	-2.85381833	-1.00208835
H	-4.09147413	-1.56689065	-2.16114723
H	-5.57226189	3.00733649	0.07773253
H	-5.53306028	2.65002600	-1.67178141
H	-5.81882229	-3.37130542	0.51008463
H	-7.01829804	2.39146920	-0.74353785
C	-8.47935907	-3.64715894	0.66126990
H	-8.38844324	0.85410232	-0.28944665
H	-9.57082366	-1.25847796	0.19739080
N	-9.05511127	-4.63303662	0.88920397
O	1.11350730	-2.75363613	0.72532818
H	1.51384480	-3.48289448	1.21949523
H	0.20129167	-2.67981205	1.04033989

3. Spectroscopic data for new compounds

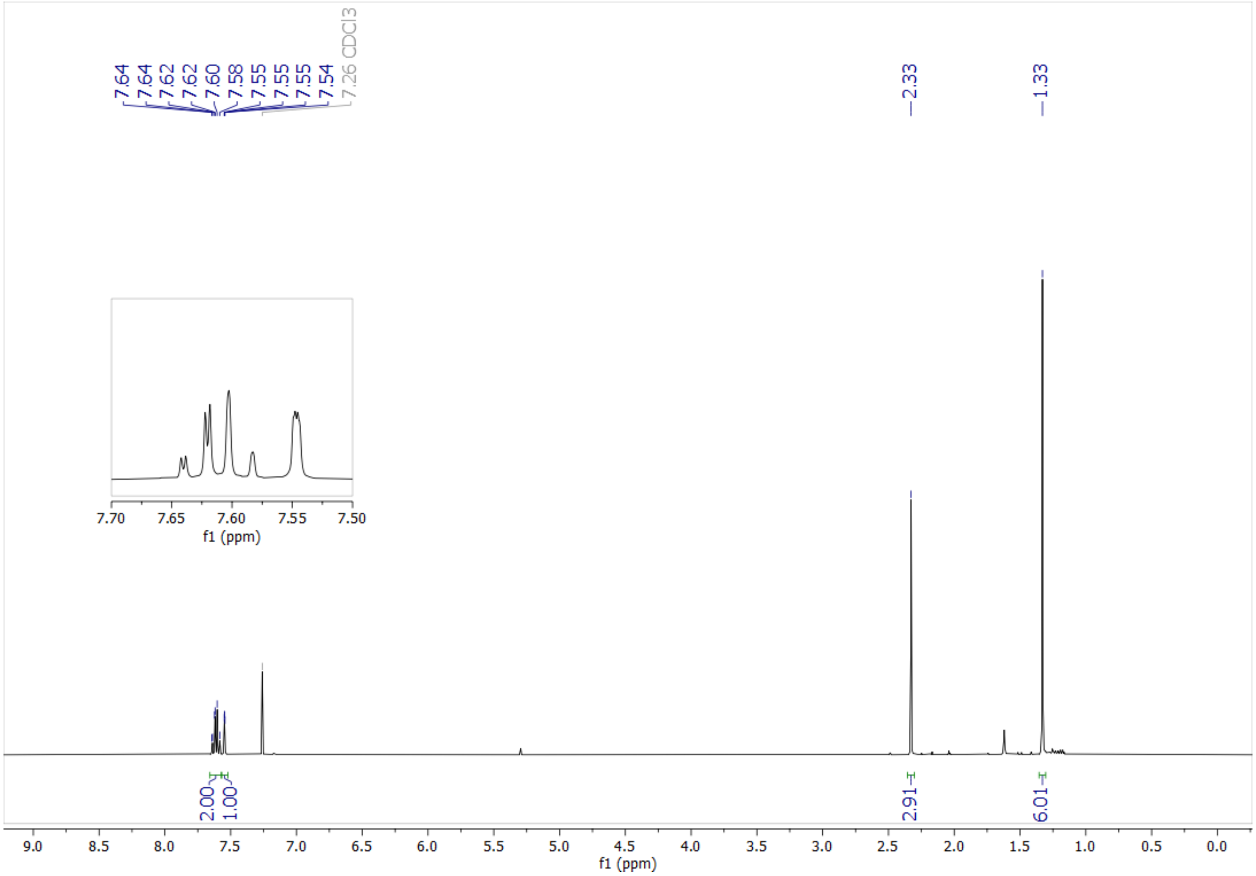

]

Figure S14. ^{1}H NMR spectrum of 2,3,3-trimethyl-3H-indole-5-carbonitrile.

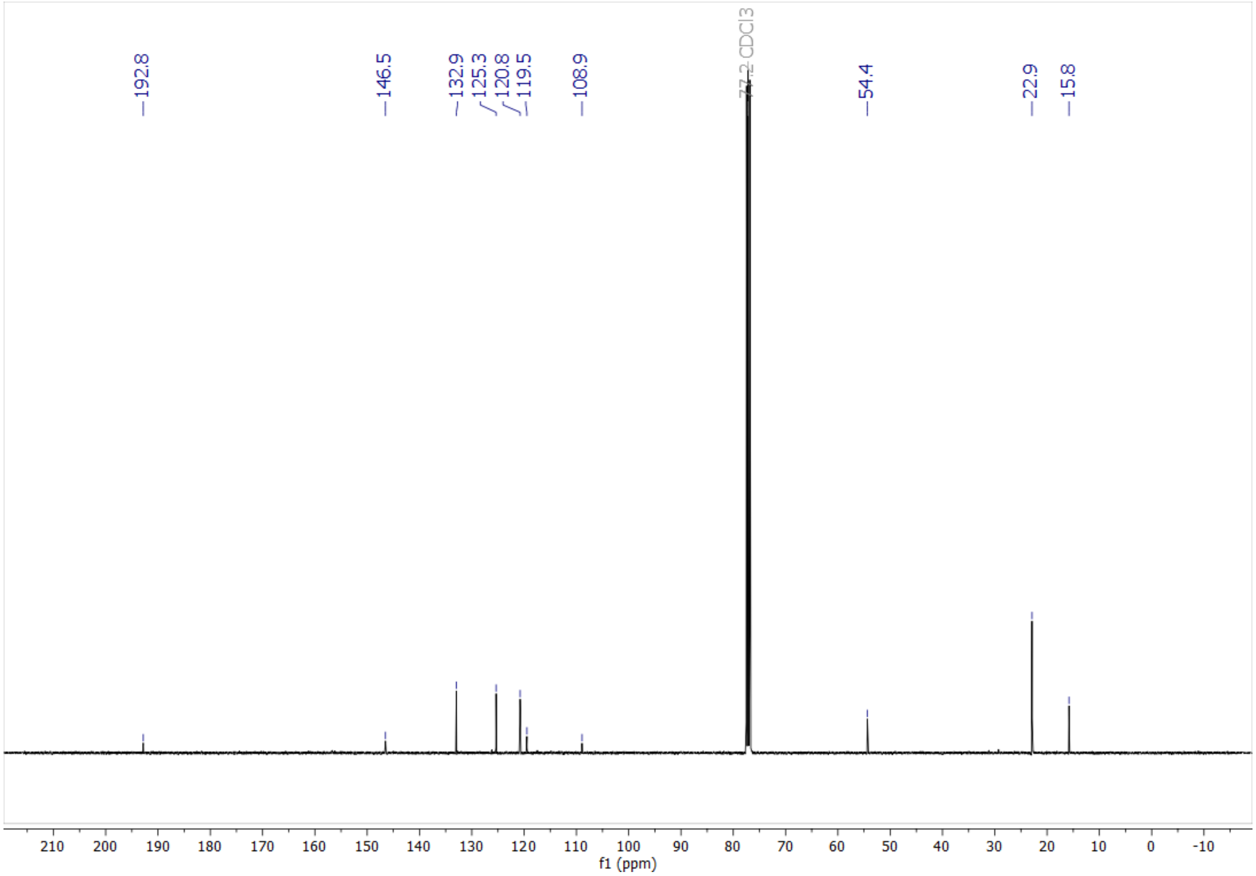

Figure S15. $^{13}C\{^1H\}$ NMR spectrum of 2,3,3-trimethyl-3H-indole-5-carbonitrile.

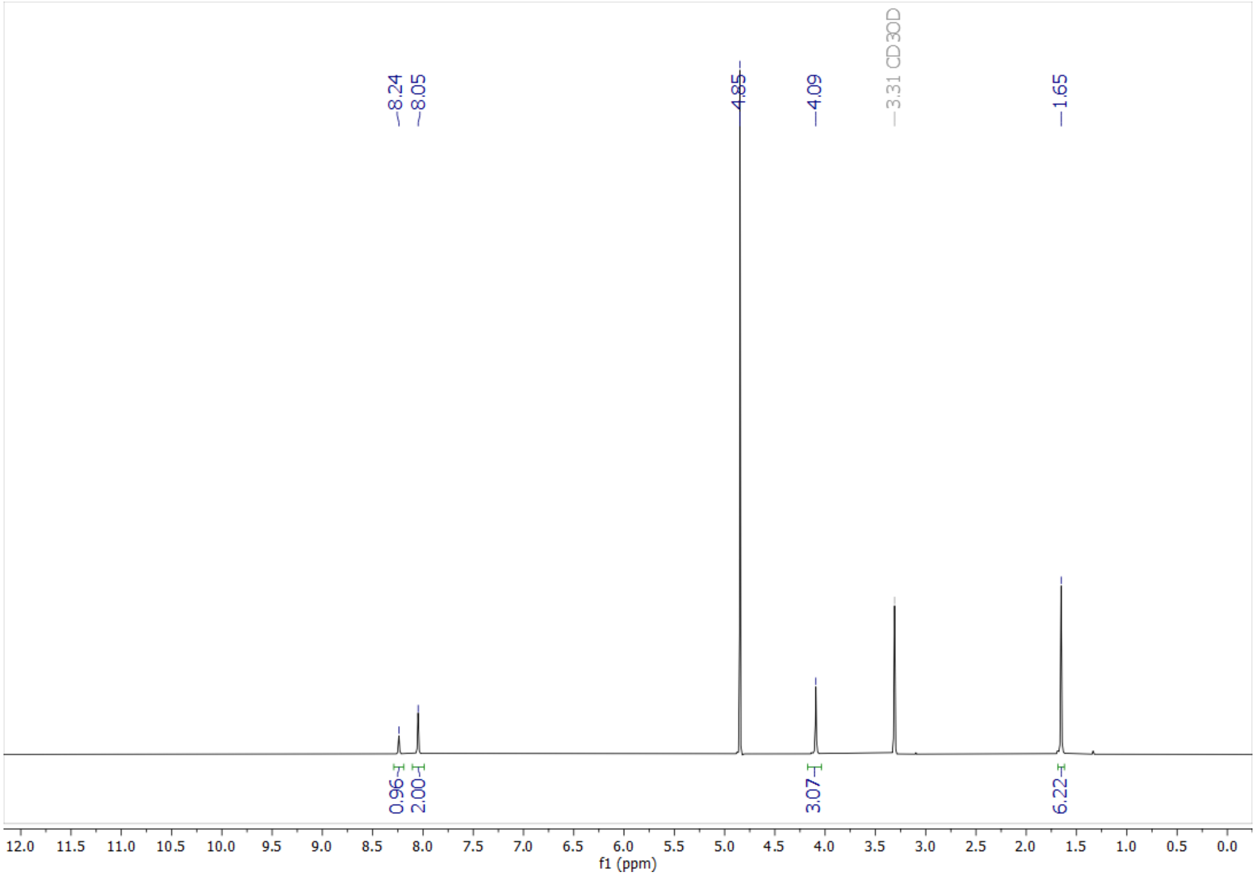

Figure S16. ^{1}H NMR spectrum of 5-cyano-1,2,3,3-tetramethyl-3H-indol-1-ium iodide.

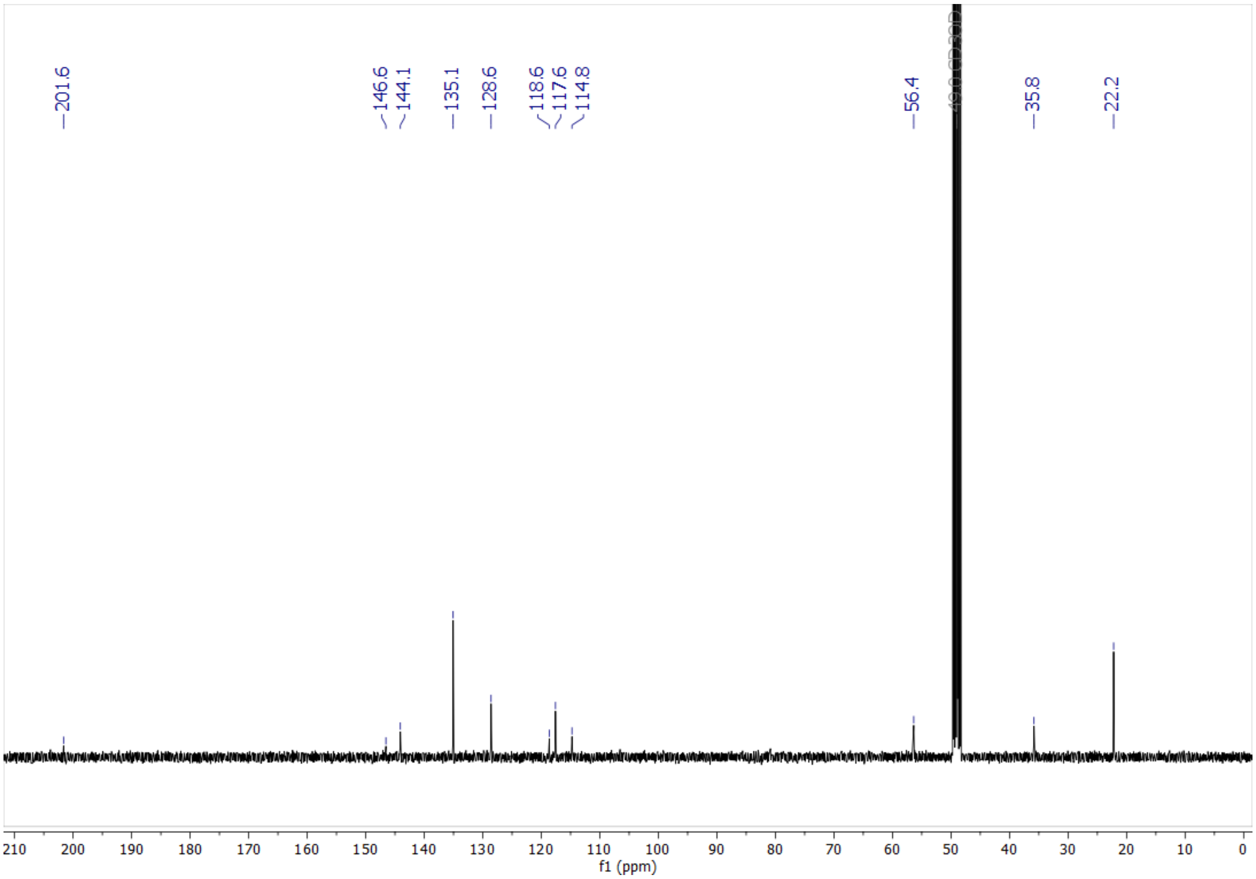

Figure S17. $^{13}C\{^{1}H\}$ NMR spectrum of 5-cyano-1,2,3,3-tetramethyl-3H-indol-1-ium iodide.

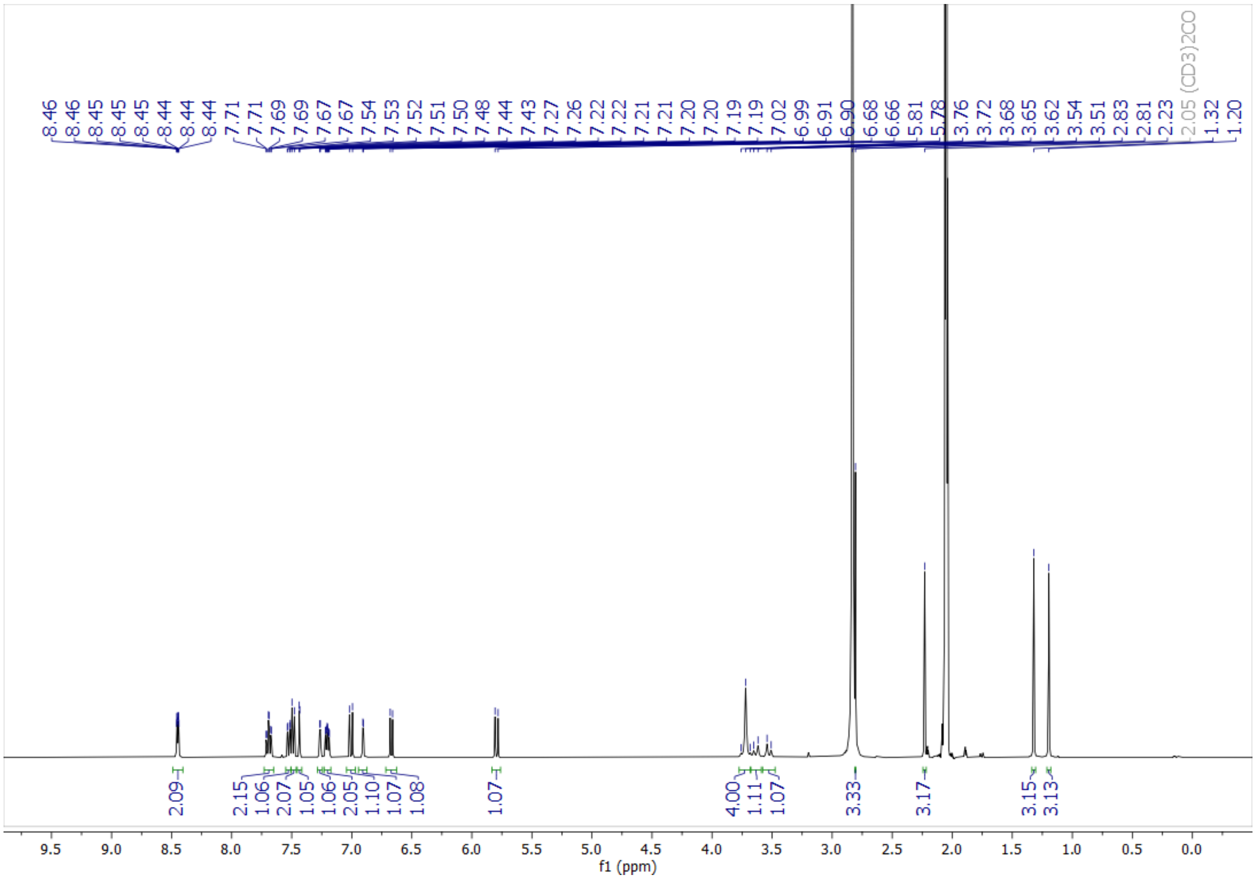

Figure S18. ^{1}H NMR spectrum of CSZin.

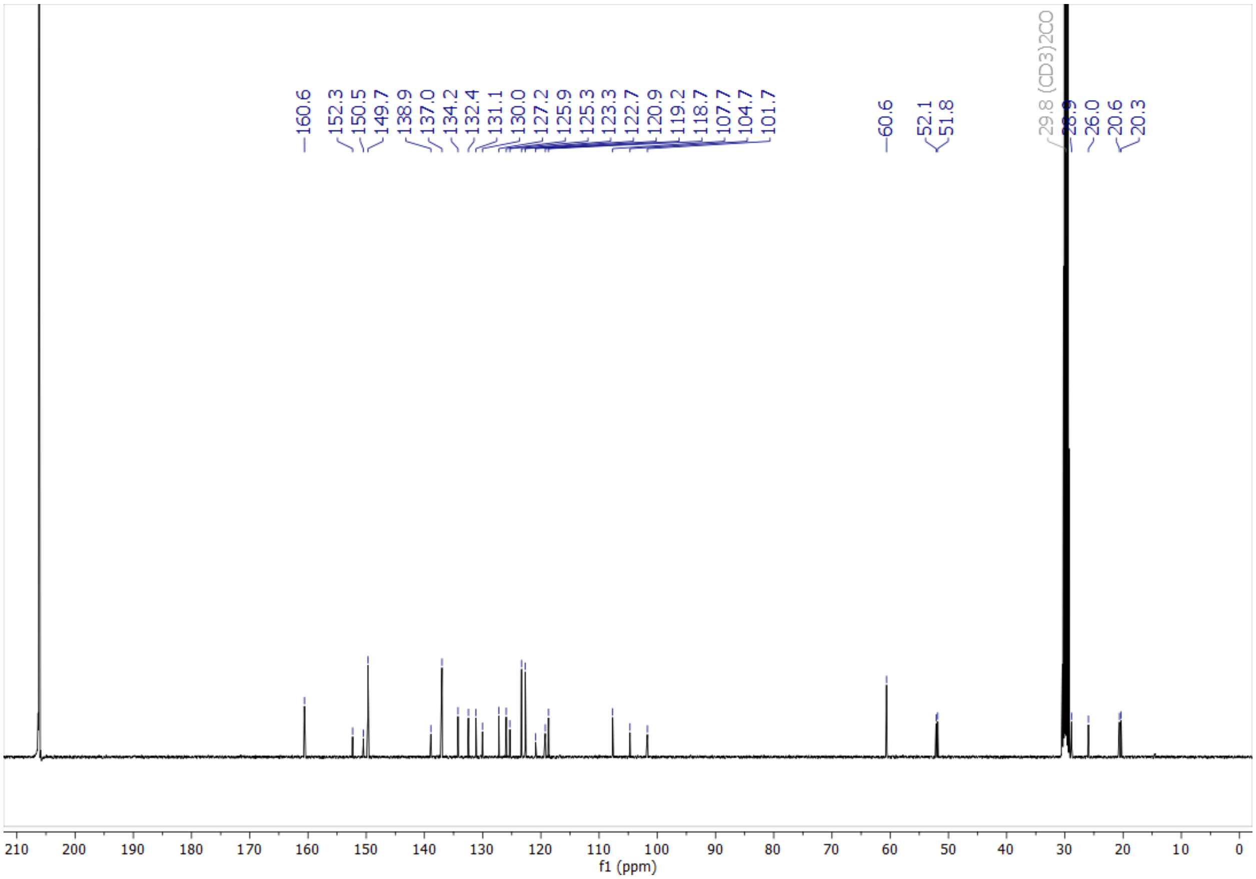

Figure S19. $^{13}C\{^1H\}$ NMR spectrum of CSZin.

4. References

1. Z. Wei, H. L. Bi, Y. Q. Liu, H. F. Nie, L. Yao, S. Z. Wang, et al., Design, synthesis and evaluation of new classes of nonquaternary reactivators for acetylcholinesterase inhibited by organophosphates, *Bioorg. Chem.*, 2018, **81**, 681-688.
2. J. Schindelin, I. Arganda-Carreras, E. Frise, V. Kaynig, M. Longair, T. Pietzsch, et al., Fiji: an open-source platform for biological-image analysis, *Nat. Methods*, 2012, **9**, 676-682.
3. K. Komatsu, Y. Urano, H. Kojima and T. Nagano, Development of an Iminocoumarin-Based Zinc Sensor Suitable for Ratiometric Fluorescence Imaging of Neuronal Zinc, *J. Am. Chem. Soc.*, 2007, **129**, 13447-13454.
4. K. K. Irikura, R. D. Johnson and R. N. Kacker, Uncertainties in Scaling Factors for ab Initio Vibrational Frequencies, *J. Phys. Chem. A*, 2005, **109**, 8430-8437.
